# The Regulatory Role of Iron Transporter SLC39A13 in Liver Fibrosis

**DOI:** 10.1002/advs.202516446

**Published:** 2026-02-04

**Authors:** Shanshan Guo, Yalin Wang, Binyu Lu, Yu Zhang, David M. Frazer, Bing Zhou

**Affiliations:** ^1^ Faculty of Synthetic Biology Shenzhen University of Advanced Technology Shenzhen China; ^2^ Key Laboratory of Quantitative Synthetic Biology, Shenzhen Institute of Synthetic Biology, Shenzhen Institutes of Advanced Technology Chinese Academy of Sciences Shenzhen China; ^3^ Molecular Nutrition Laboratory QIMR Berghofer Medical Research Institute Herston Australia

**Keywords:** collagen, hepatic stellate cells, iron, liver fibrosis, SLC39A13 (ZIP13), VA‐lip‐siRNA

## Abstract

Liver fibrosis, driven by excessive collagen synthesis following hepatic injury, poses a significant health challenge. SLC39A13/ZIP13, a recently characterized intracellular iron transporter, is shown to provide iron to the ER/Golgi to help catalyze procollagen hydroxylation during collagen maturation. Here, we investigate whether ZIP13 plays a role during hepatic fibrogenesis modeled by CCl_4_ stress or other inducers. ZIP13 expression is induced during liver fibrosis. Germline disruption of *Zip13* dramatically reduces fibrosis. Surprisingly, these mice do not benefit from ZIP13 loss after CCl_4_ challenge; instead, they are more susceptible to CCl_4_ toxicity even with substantially less fibrosis development. This elevated vulnerability turns out to be a consequence of ferroptosis in the hepatocyte due to increased cytosolic iron after ZIP13 loss. Tissue‐specific knockout (KO) reveals that hepatic stellate cell (HSC) KO of *Zip13* attenuates liver fibrosis progression without adverse effects. Leveraging these findings, HSC‐targeted delivery of *Zip13*‐siRNA demonstrates robust efficacy and safety in preclinical fibrosis models. These results provide critical insights into the complex role of iron in liver fibrosis, and indicate that targeting iron homeostasis via ZIP13 in the HSC may be effective to mitigate fibrogenesis by simultaneously suppressing the synthesis of multiple kinds of collagen while minimizing possible side effects.

## Graphical abstract

SLC39A13/ZIP13, a newly discovered intracellular iron transporter, delivers iron to the ER/Golgi to catalyze procollagen hydroxylation during collagen maturation. Here, we systematically characterize the cell type‐specific functions of ZIP13 across distinct hepatic cell populations, and identify hepatic stellate cell‐specific ZIP13 as a promising and safe therapeutic target for the suppression of liver fibrosis.

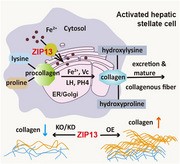

**Abbreviations**
aHSCs: activated hepatic stellate cells
ALT: alanine aminotransferase
ALD: alcohol‐associated liver disease
AST: aspartate aminotransferase
CCl_4_
: carbon tetrachloride
CLDs: chronic liver diseases
CP4H: collagen prolyl 4‐hydroxylase
Col I: type I collagen
Col III: type III collagen
Col IV: type IV collagen
DFP: deferiprone
ECM: extracellular matrix
fer‐1: ferrostatin‐1
HSCs: hepatic stellate cells
LSECs: liver sinusoidal endothelial cells
ir: immunoreactivity
IL‐1β: interleukin‐1 β
KO: knockout
LH: lysyl hydroxylase
MASH: metabolic dysfunction‐associated steatohepatitis
MCD diet: methionine‐ and choline‐deficient diet
MASLD: metabolic dysfunction‐associated fatty liver disease
NF‐κB: nuclear factor kappa‐light‐chain‐enhancer of activated B cells
OE: overexpression
SCD‐EDS: spondylocheiro dysplastic Ehlers‐Danlos syndrome (EDSSPD3, OMIM #612350)
TGF‐β: transforming growth factor‐beta
IL‐1β: interleukin‐1 βeta
WT: wild‐type
*Zip13* ^−^ *^/^* ^−^
: systemic/whole‐body (germline) deletion of *Zip13*
*Zip13^fl/fl^*
: *Zip13* conditional knockout (with loxp sites)
*Zip13 OE^fl/fl^*
: *Zip13* conditional overexpression (conditional knock‐in, with loxp sites)
*Zip13^LratL/rat^*
: *Zip13* knockout in hepatic stellate cells, generated by intercrossing the offspring of *Lrat‐Cre* and *Zip13^fl/fl^* mice
*Zip13^Alb/Alb^*
: *Zip13* knockout in hepatocytes, generated by intercrossing offspring of *Alb‐Cre* and *Zip13^fl/fl^* mice
*Zip13^Tie−2/Tie−2^*
: *Zip13* knockout in liver sinusoidal endothelial cells, generated by intercrossing the offspring of *Tie‐2‐Cre* and *Zip13^fl/fl^* mice
*Rosa‐Cre^+/^* ^−^ *Zip13 OE^+/^* ^−^
: *Zip13* systemic/whole‐body overexpression, generated by crossing the *Rosa‐Cre* and *Zip13 OE^fl/fl^* mice
*Zip13OE^LratL/rat^*
: *Zip13* overexpressed in hepatic stellate cells, generated by intercrossing the offspring of *Lrat‐Cre* and *Zip13OE^fl/fl^* mice

## 1 Introduction

According to estimates from the 2019 Global Burden of Disease study, fibroproliferative disorders account for approximately 35.4% of all global mortality [1], highlighting a substantial unmet medical need. This urgency underscores the imperative to develop effective therapies capable of halting fibrosis progression or, ideally, promoting its resolution [2]. Liver fibrosis, the most prevalent fibroproliferative disorder, represents a pathological wound‐healing response to chronic liver injury. Its core pathological features include excessive deposition of extracellular matrix (ECM) components and disruption of normal hepatic architecture. In a study of the general population, vibration‐controlled transient elastography (VCTE) data indicate a global prevalence of 7.3% for significant liver fibrosis, 3.5% for advanced fibrosis, and 1.2% for cirrhosis [3]. Liver fibrosis critically drives the progression of diverse chronic liver diseases (CLDs), such as viral hepatitis, alcohol‐associated liver disease (ALD), and metabolic dysfunction‐associated steatotic liver disease (MASLD), often advancing to cirrhosis and hepatic failure [4]. Despite etiological differences among CLDs, liver fibrosis and cirrhosis represent a common pathological endpoint [5].

Recent research on fibrosis has focused on elucidating its molecular mechanisms, clarifying immune‐regulatory processes, and identifying potential strategies for its reversal [6]. Key therapeutic approaches under investigation include targeting hepatic stellate cells (HSCs), modulating inflammatory signaling pathways (e.g., TGF‐β and NF‐κB) [7], and promoting extracellular matrix (ECM) degradation to halt or reverse fibrogenesis [2, 4, 8]. Furthermore, nanotechnology‐based drug delivery systems [9, 10] and gene editing technologies (e.g., CRISPR‐Cas9) [11] have shown promise in the treatment of liver fibrosis. However, few clinically approved anti‐fibrotic agents currently exist [12], underscoring the critical need to develop safer and more effective therapeutic targets and modalities against liver fibrosis.

HSC activation is a central mediator of hepatic fibrogenesis [13, 14]. In healthy liver tissue, HSCs remain quiescent and function as primary storage sites for vitamin A. Following chronic injury stimuli, including viral hepatitis, ALD, and MASLD, HSCs undergo activation and differentiate into myofibroblast‐like cells [13, 15]. Activated HSCs (aHSCs) subsequently synthesize and secrete abundant ECM components, predominantly collagens I and III, driving fibrotic scar deposition [16]. Progressive ECM accumulation disrupts the native tissue architecture, impairing hepatocyte function and compromising microvascular perfusion [17].

Collagens constitute the dominant protein components of the ECM. In the fibrotic liver, aHSCs are primarily responsible for synthesizing interstitial ECM proteins, notably type I, III, V, and VI collagens (Col I, III, V, and VI) [17]. While aHSCs also produce type IV collagen (Col IV), this collagen subtype is additionally synthesized by epithelial and endothelial cells. Unlike the fibrillar collagens (e.g., types I and III) secreted by fibroblasts or aHSCs, Col IV is a network‐forming collagen with a distinct, looser organizational architecture [18]. Despite these structural variations, all collagen types share a characteristic triple‐helical structure stabilized by three polypeptide chains containing repetitive (Gly‐X‐Y)n sequences, where X and Y are frequently proline and hydroxyproline residues [19]. The Hydroxylated proline and lysine is critical for establishing the compact, stable conformation of collagen, rendering it resistant to degradation within the ECM [20]. This post‐translational modification—catalyzed by collagen prolyl 4‐hydroxylases (CP4Hs) or lysyl hydroxylases (LHs) in the endoplasmic reticulum (ER)/Golgi—requires sufficient Fe^2^^+^ as a cofactor [21, 22].

Our laboratory identified ZIP13 (SLC39A13) as the exclusive mammalian transporter responsible for cytosolic‐to‐ER/Golgi iron flux. This molecular role is consistent with phenotypes observed in *Zip13^‑/‑^* murine models and in patients with spondylocheiro dysplastic Ehlers‐Danlos syndrome (SCD‐EDS/EDSSPD3, OMIM #612350) harboring *ZIP13* mutations [23, 24, 25]. These findings indicate that compartmental iron deficiency in the ER/Golgi disrupts collagen cross‐linking, maturation, and secretion. In healthy tissues, the precise stacking of procollagen molecules forms a solid structure resistant to degradation, contributing to an ECM rigid enough to provide structural support. In fibrotic liver, excessive cross‐linked collagen forms persistent “scars” that are difficult to resolve, driving pathological progression toward conditions like cirrhosis and hepatocellular carcinoma. In contrast, non‐hydroxylated procollagen is rapidly cleared following its secretion from aHSCs [21]. Consistently, CP4H has been demonstrated as a promising therapeutic target for anti‐fibrotic intervention [21]. We wondered whether ZIP13, a functional partner of CP4H, might also serve a similar role.

Although ZIP13 is a widely expressed metal transporter, hepatic ZIP13 is more abundant in the mesenchymal cells (including HSCs) than in hepatocytes, Kupffer cells, and endothelial cells, based on human and mouse protein atlas data. This suggests that ZIP13 may perform specific functions in these cell types (Figure S1A,B) [26, 27]. Given that HSCs synthesize and secrete collagen upon activation, targeted intervention against collagen biosynthetic pathways in activated HSCs may constitute a safe and efficacious therapeutic strategy. Such an approach would circumvent unintended impairment of quiescent HSC functions and avoid disruption of physiological collagen synthesis in other tissues. In the present study, we investigated the role of ZIP13 in liver fibrosis, and identified the HSC‐expressed ZIP13 as a promising therapeutic target to interfere with the maturation of multiple collagen subtypes and to halt the progression of liver fibrosis.

## 2 Results

### 2.1 ZIP13 Deficiency Suppresses Collagen Formation during Liver Fibrosis

**FIGURE 1:**
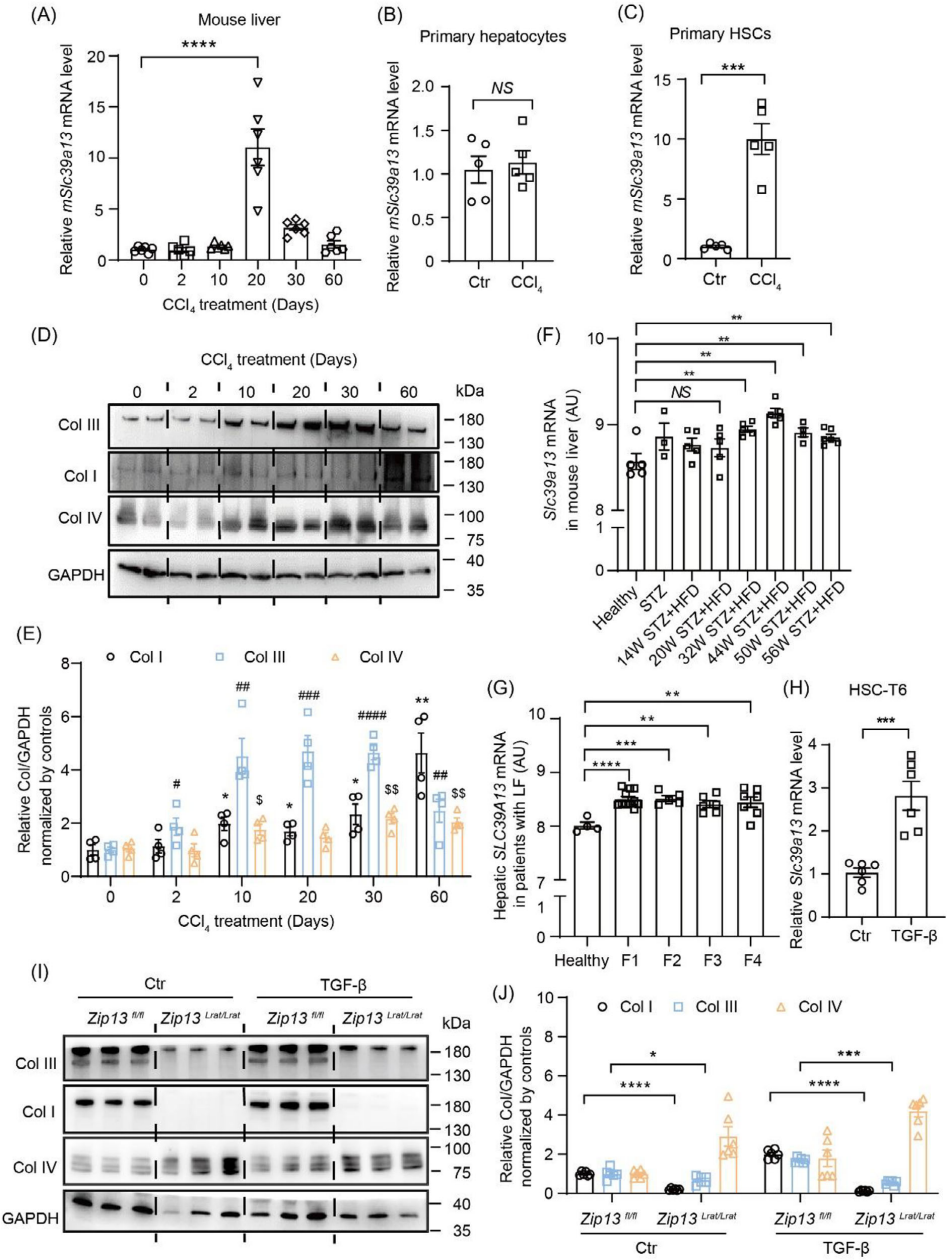
ZIP13 was induced during fibrosis and its deficiency suppressed collagen formation. (A–D) mRNA levels of *Zip13* (*Slc39a13*) in (A) the liver from the mice with CCl_4_ administration for different days (n = 6), primary (B) hepatocytes and (C) hepatic stellate cells isolated from the liver of mice with CCl_4_ administration for 20 days (n = 5). (D,E) levels of type I collagen (Col I), type III collagen (Col III) and type IV collagen (Col IV) in the liver by western blotting after CCl_4_ administration for different days (n = 4). Data are shown as the mean ± SEM. *, *P* < 0.05; **, *P* < 0.01; Col I in other groups *VS* 0‐day group. #, *P* < 0.05; ##, *P* < 0.01; ###, *P* < 0.001; ####, *P* < 0.01; Col III in other groups *VS* 0‐day group. $, *P* < 0.05; $$, *P* < 0.01; Col IV in other groups *VS* 0‐day group. Statistical analysis was performed separately for Col I, Col III and Col IV by the two‐sided Student's t‐test. (F) *Zip13* (*Slc39a13*) mRNA levels in a streptozotocin (STZ) combined with high‐fat diet (HFD) ‐induced mouse model at multiple time points: at 32 weeks when MASH with fibrosis developed, at 44 weeks with progression to MASH with advanced fibrosis, and at 50–56 weeks when MASLD‐related dysplastic nodules and hepatocellular carcinoma emerged (GSE246221). For Healthy controls, 14W STZ+HFD, 20W STZ+HFD, 32W STZ+HFD, and 44W STZ+HFD groups, n = 5; for STZ group, n = 3; for 50W STZ+HFD group, n = 4; for 56W STZ+HFD group, n = 6. (G) *ZIP13* (*SLC39A13*) mRNA expression in patients with liver fibrosis classified as METAVIR stages F1‐F4 (GSE246221). Healthy controls, n = 4; F1 group, n = 11; F2 group, n = 5; F3 group, n = 6; F4 group, n = 7. (F,G) was generated from the sequencing data published by Jeong BK, et al. in 2024 [23]. (H) mRNA levels of *Zip13* (*Slc39a13*) in hepatic stellate cell line (HSC‐T6) activated with TGF‐β (10 ng/mL) for 24 h (n = 6). (I,J) Western blotting for Col I, Col III, Col IV, and GAPDH in wild‐type (*Zip13^fl/fl^*) and *Zip13^Lrat/Lrat^* immortalized HSCs induced by TGF‐β (10 ng/mL) for 24 h. n = 6. In (A–C,F–H,J) data are shown as the mean±SEM. *NS*, no significant; *P* < 0.05; **, *P* < 0.01; ***, *P* < 0.001; ****, *P* < 0.0001. Statistical analysis in panel A was performed by the one‐way ANOVA with multiple comparisons; in (B,C,H,J) by the two‐sided Student's t‐test. For RNA sequencing data in panels (F,G), statistical analysis was performed using the algorithm of Benjamini & Hochberg test.

To explore the role of ZIP13 in liver fibrosis pathogenesis, we first investigated whether ZIP13 expression would alter during progressive fibrosis stages in a CCl_4_‐induced mouse model of fibrosis (Figure 1A). Interestingly, *Zip13* mRNA levels increased sharply about 10 to 20 days after CCl_4_ administration (25% v/v, twice weekly by oral gavage), subsequently declining to 3–5 folds above baseline after 30–60 days of treatment (Figure 1A). To specifically analyze whether HSCs and hepatic parenchymal cells (hepatocytes) were affected, we isolated these cells from mice treated with CCl_4_ for 20 days. Compared to their respective controls from untreated mice, the mRNA levels of *Zip13* were significantly elevated in HSCs but remained unchanged in hepatocytes (Figure 1B,C). Furthermore, according to the public database, high enrichment of *ZIP13* in HSCs was found in both healthy humans and mice, implicating that ZIP13 may be involved in functions related to these cells (Figure S1A,B).

Levels of Col III and Col IV altered following a roughly similar pattern of *Zip13* mRNA changes over time (Col IV expression started slightly earlier), while Col I lagged relatively behind (Figure 1D,E). These results suggest a correlation between ZIP13 expression and collagen synthesis during the pathogenic process. Notably, mining the sequencing data from Jeong BK, et al. [28] also revealed increase of hepatic *Slc39a13* (*Zip13*) mRNA in the livers of mouse models induced with streptozotocin (STZ) plus high‐fat diet (HFD) (Figure 1F), as well as in the liver of patients with liver fibrosis at F1–F4 stages compared with healthy controls (Figure 1G).

During liver fibrosis, inflammation activates HSCs to synthesize and secrete collagens. In vitro, immortalized WT HSCs after TGF‐β stimulation recapitulate this process, also resulting in a significant increase in collagen production compared to their quiescent counterparts. We, therefore, first investigated in vitro whether ZIP13, which provides the necessary iron for collagen maturation, participates in this process. Stimulation of HSC‐T6 cells with TGF‐β led to a marked increase in *Zip13* mRNA level (Figure 1H). As anticipated, the increase of collagen failed to happen in *Zip13*‐deficent HSCs, regardless of TGF‐β stimulation (Figures 1I,J; S2A–D).

**FIGURE 2:**
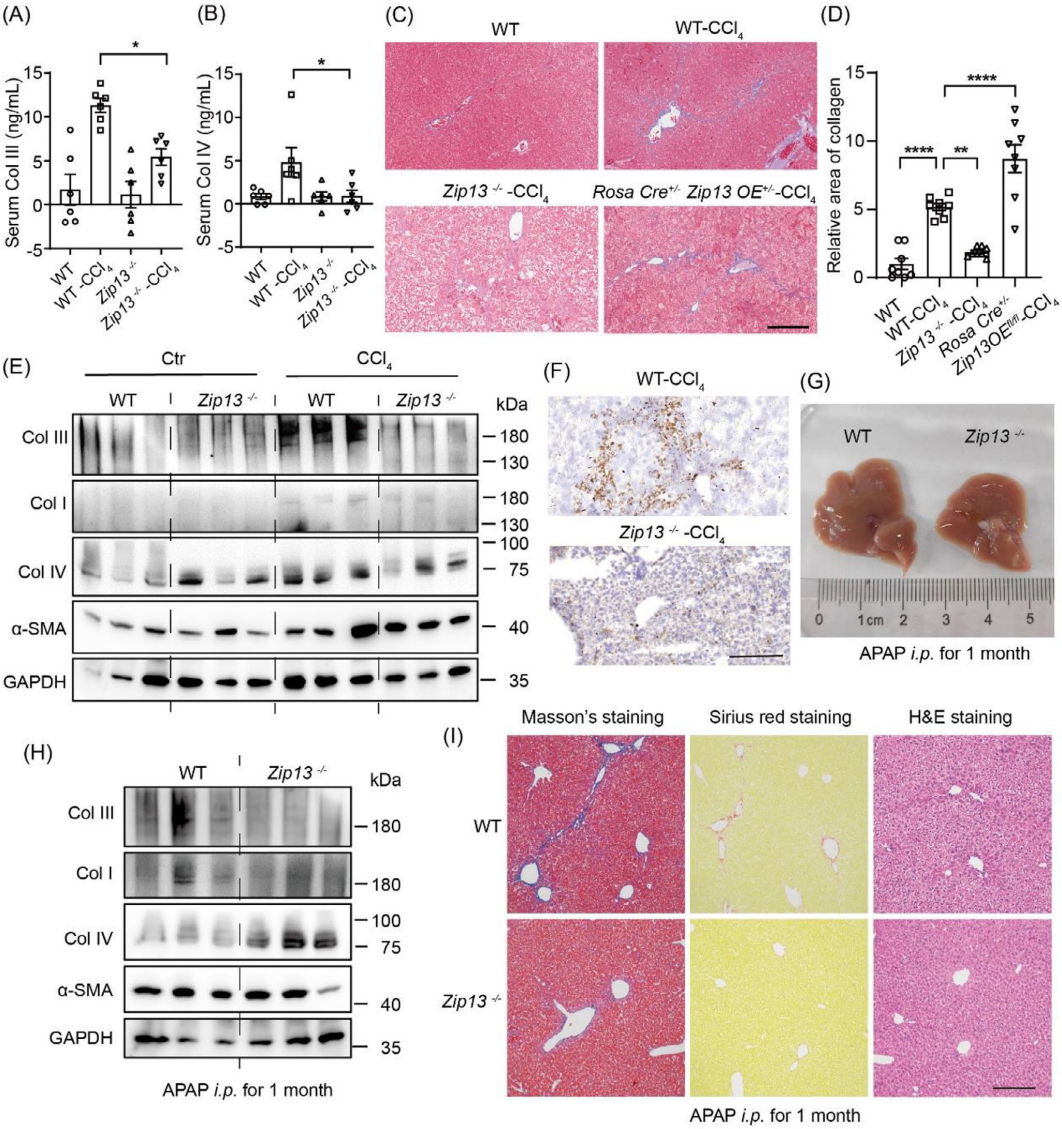
ZIP13 deficiency suppressed progression of liver fibrosis in mice. Serum (A) Col III, (B) Col IV of the WT and *Zip13* ^−^ *^/^* ^−^ mice with and without CCl_4_ induction for 1 month, n = 6. (C, D) Masson's trichrome staining of liver from WT*, Zip13* ^−^ *^/^* ^−^, and *Zip13 OE* (*Rosa‐Cre ^+/^* ^−^ *Zip13OE^+/^* ^−^) male mice after 1 month of CCl_4_ treatment, n = 8. (E) Western blotting for Col I,Col III, Col IV, α‐smooth muscle actin (α‐SMA), and GAPDH in liver of WT and *Zip13* ^−^ *^/^* ^−^ mice with and without CCl_4_ treatment for 1 month. (F) Immuno‐histochemical staining (enhanced by DAB) for Col I in the liver of WT and *Zip13* ^−^ *^/^* ^−^ mice following CCl_4_ treatment for 1 month. (G) Gross appearance, (H) Western blotting images for Col I, Col III, Col IV, α‐SMA, and GAPDH, and (I) H&E, Masson's, and Sirius red staining of the liver from WT and *Zip13* ^−^ *^/^* ^−^ mice intraperitoneally injected with acetaminophen (APAP, 250 mg/kg, every 2 days) for 1 month. Scale bars, 200 µm in (C,F,I). In (A,B,D) data are shown as the mean±SEM. *, *P* < 0.05; **, *P* < 0.01; ****, *P* < 0.0001. Statistical analysis in panels (A,B,D) was performed by the one‐way ANOVA with multiple comparisons.

We next investigated in vivo the functional link between ZIP13 and hepatic collagen deposition. We used *Zip13* ^−/−^ mice (germline *Zip13* knockout, Figure S2E) to analyze if ZIP13 deficiency would inhibit collagen deposition during liver fibrosis. Following 1 month of CCl_4_ treatment, *Zip13* ^−^ *^/^* ^−^ mice demonstrated significantly reduced serum type III procollagen (Figure 2A), Col IV (Figure 2B), and hepatic collagen deposition (Figures 2C–F; S3A) compared to WT controls. Interestingly, lamin, another fibrotic marker and a non‐collagenous structural protein, also decreased in *Zip13* ^−^ *^/^* ^−^ mice, whereas hyaluronic acid did not show this trend (Figure S3B,C). Conversely, ZIP13 overexpression (*Zip13 OE* or more specifically, *Rosa‐Cre^+/^* ^−^ *Zip13OE ^+/^* ^−^) mice exhibited markedly increased collagen accumulation (Figure 2C,D). To address whether ZIP13's role is specific to the CCl_4_ fibrosis model or not, we additionally established the acetaminophen (APAP)‐induced mouse model of liver fibrosis. In the APAP liver fibrosis model, livers from *Zip13* ^−^ *^/^* ^−^ mice displayed less stiffness (Figure 2G) and less collagen deposition (Figures 2H,I; S3D). Notably, ZIP13 dysregulation affected levels of several major types of collagens, including Col I, Col III and Col IV (Figures 1D,E,I,J and 2A,B,E,H; S4), indicating that ZIP13 mediates broadly collagen synthesis or stability.

### 2.2 ZIP13 Deficiency in Hepatocytes Exacerbates CCl_4_‐Induced Hepatotoxicity via Iron Elevation

**FIGURE 3:**
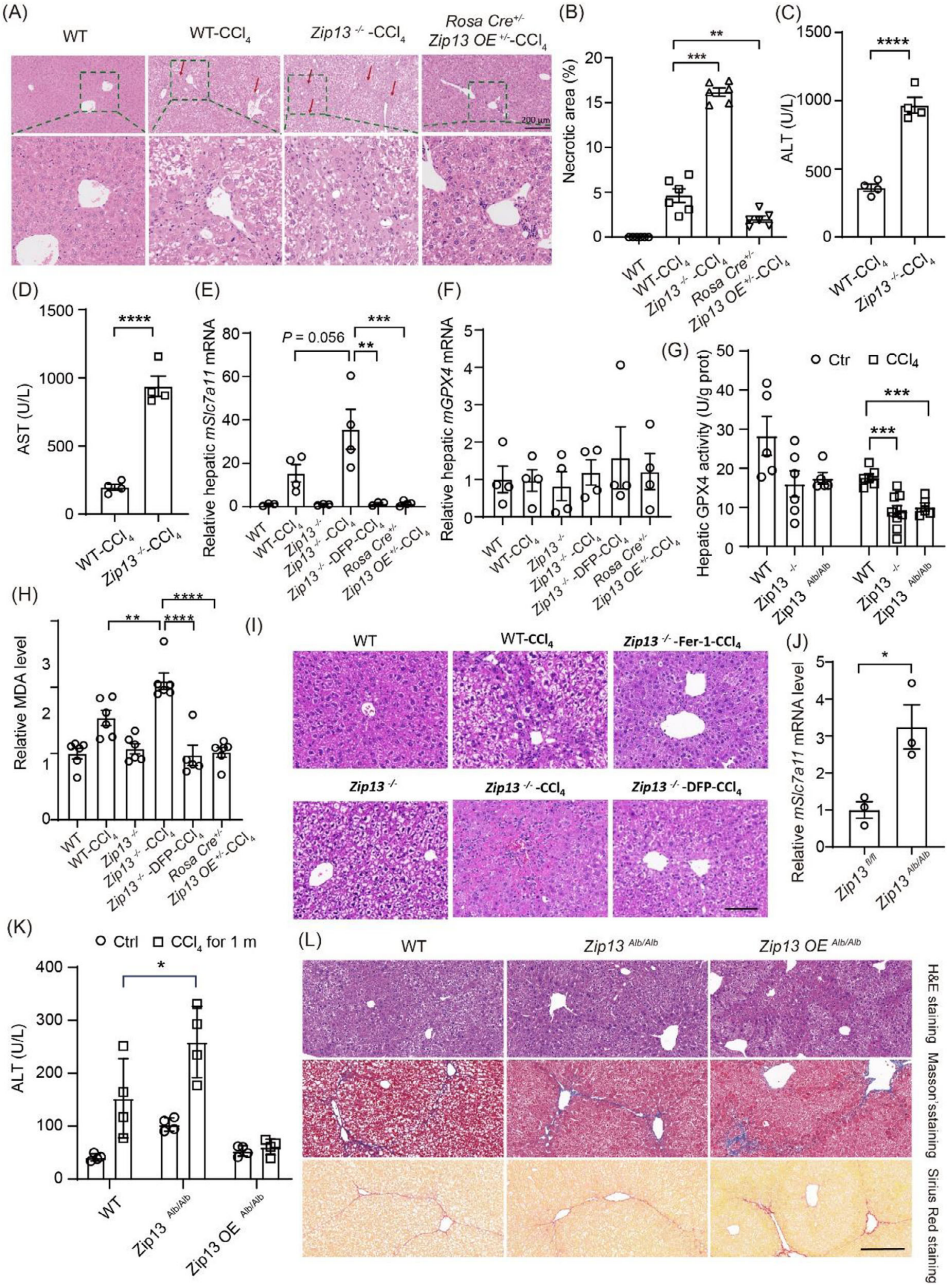
ZIP13 deficiency exacerbated CCl_4_‐liver toxicity via iron dyshomeostasis in hepatocytes. (A) H&E staining of the liver from WT*, Zip13* ^−^ *^/^* ^−^, and *Rosa‐Cre ^+/^* ^−^ *Zip13OE ^+/^* ^−^ (*Zip13* overexpression throughout the body) male mice after short‐term (1 month) of CCl_4_ treatment. (B) Quantitation of the necrotic area in livers from WT*, Zip13* ^−^ *^/^* ^−^, and *Zip13 OE* (overexpression throughout the body) male mice after 1 month of CCl_4_ treatment, n = 6. (C,D) Serum alanine aminotransferase (ALT) and aspartate aminotransferase (AST) levels of WT and *Zip13* ^−^ *^/^* ^−^ male mice after 1 month of CCl_4_ treatment, n = 4. (E,F) Relative mRNA levels of *Slc7a11* and *Gpx4* in livers from WT mice and *Zip13* ^−^ *^/^* ^−^ mice both with and without CCl_4_ treatment for 1 month and *Zip13 OE* (overexpression throughout the body) mice treated with CCl_4_ for 1 month. Deferiprone (DFP ‐ 50 mg/kg, twice a week) was also intraperitoneally injected in a separate cohort of CCl_4_‐treated *Zip13* ^−^ *^/^* ^−^ mice. n = 4. (G) Relative hepatic GPX4 activity in WT, *Zip13* ^−^ *^/^* ^−^ and *Zip13^Alb/Alb^* (*Zip13* knockout in hepatocytes) mice with and without CCl_4_ treatment for 1 month, n = 6. (H) MDA levels (n = 6) and (I) H&E staining of the livers from WT mice and *Zip13* ^−^ *^/^* ^−^ mice both with and without CCl_4_ treatment for 1 month, and *Zip13 OE* (in whole body, *Rosa‐Cre ^+/^* ^−^ *Zip13OE ^+/^* ^−^) mice treated with CCl_4_ for 1 month. Deferiprone (DFP ‐ 50 mg/kg, twice a week) was also intraperitoneally injected into a separate cohort of CCl_4_‐treated *Zip13* ^−^ *^/^* ^−^ mice in (E–H), and ferrostain‐1 (Fer‐1, 5 mg/kg, twice a week) was intraperitoneally injected for 1 month during CCl_4_ treatment in I. (J) Relative hepatic mRNA levels of *Slc7a11* (n = 3), (K) serum ALT (n = 4), and (L) H&E, Masson's and Sirius red staining of the liver of WT, *Zip13^Alb/Alb^* (*Zip13* knockout in hepatocytes) and *ZIP13 OE^Alb/Alb^* (*Zip13* overexpression in hepatocytes) mice treated for 1 month with CCl_4_ gavage. Scale bars, 200 µm in (A,L), 100 µm in (I). In (B–H,J,K) data are shown as the mean±SEM. *, *P* < 0.05; **, *P* < 0.01; ***, *P* < 0.001; ****, *P* < 0.0001. Statistical analysis in panels (B,E,F,H) was performed by the one‐way ANOVA with multiple comparisons; in (C,D,G,J) by the two‐sided Student's t‐test, and in panel (K) by the two‐way ANOVA with multiple comparisons.

Paradoxically, after CCl_4_ treatment, while *Zip13* ^−^ *^/^* ^−^ mice exhibited reduced hepatic collagen deposition, both histopathological analyses of liver sections and elevated serum ALT/AST levels indicated more severe liver injury compared to WT controls (Figure 3A–D). These mice were generally less healthy despite reduced fibrosis. Given ZIP13's established role in iron homeostasis, we investigated whether iron alteration contributes to the exacerbated CCl_4_‐induced injury in *Zip13* ^−^ *^/^* ^−^ livers. One specific type of iron toxicity is ferroptosis. Initial assessment revealed increased *Slc7a11* mRNA to resist oxidative stress, but no change in *Gpx4* mRNA expression, after *Zip13* loss (Figure 3E,F). However, GPX4 activity was significantly reduced in *Zip13* ^−^ *^/^* ^−^ mice compared to WT mice under CCl_4_ exposure (Figure 3G). Malondialdehyde (MDA), superoxide dismutase (SOD) activity, and reactive oxygen species (ROS) analyses suggested elevated lipid peroxidation in *Zip13* ^−/−^ versus WT mice (Figures 3H; S5A,B). Crucially, administration of either the iron chelator deferiprone (DFP) or the ferroptosis inhibitor ferrostatin‐1 (Fer‐1) effectively mitigated the enhanced liver injury in *Zip13* ^−^ *^/^* ^−^ mice, demonstrated by normalization of *Slc7a11* expression (Figure 3E), improved hepatic histology (H&E staining, Figure 3I), as well as reduced serum ALT and AST levels (Figure S5C,D). These results indicate that elevated iron, and possibly ferroptosis, is responsible for the enhanced CCl_4_ hepatotoxicity in *Zip13* ^−/−^ mice.

Hepatocytes, the predominant cell type in the liver, mediate systemic iron storage. To determine whether iron‐related liver injury resulted specifically from ZIP13 deficiency within hepatocytes, we disrupted *Zip13* in hepatocytes only (*Zip13^Alb/Alb^*) (Figures S2A,H and S5E,F). These mice exhibited decreased activity of GPX4 and elevated hepatic *Slc7a11* mRNA levels compared to littermate controls (Figure 3G,J). Consistently, significantly higher serum ALT and AST levels than those in WT mice were seen in *Zip13^Alb/Alb^* mice when treated with CCl_4_ for 1 month, whereas these levels in mice with ZIP13 overexpression in hepatocytes (*Zip13OE^Alb/Alb^*) remained comparable to that of the controls or exhibited a slight downward trend (Figures 3K; S5G). Although the injury phenotype in *Zip13^Alb/Alb^* resembled that observed in *Zip13* ^−^ *^/^* ^−^ mice, histopathological analysis revealed no significant difference in ECM collagen deposition between *Zip13^Alb/Alb^* and WT mice following 1 month of CCl_4_ exposure (Figures 3L; S5H,I). These results indicate that hepatocyte‐specific ZIP13 deficiency mediates hepatotoxicity under CCl_4_ stress but is not a significant player, either directly or indirectly, in affecting collagen levels within the hepatic ECM.

### 2.3 ZIP13 Overexpression Accelerates Liver Cirrhosis

**FIGURE 4:**
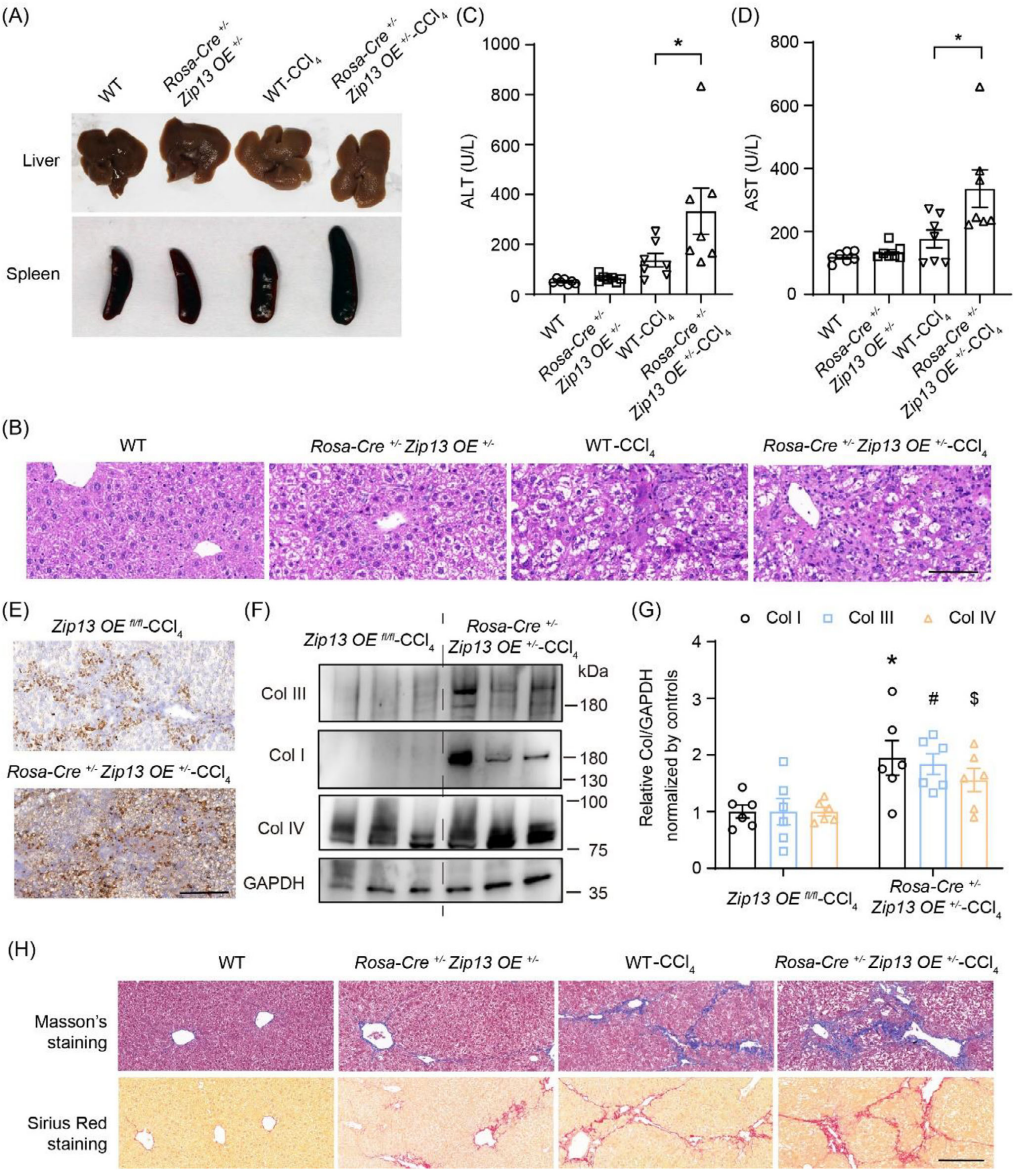
Overexpression of ZIP13 accelerated liver cirrhosis after CCl_4_ treatment. Tissues from WT and *Rosa‐Cre^+/^* ^−^ *Zip13OE^+/^* ^−^ (*Zip13* overexpression throughout the body) mice with and without treatment with CCl_4_ for long‐term (2 months) were examined. (A) Gross appearance of livers and spleens. (B) H&E staining of the liver. (C,D) Serum ALT and AST, n = 7. Data are shown as the mean ± SEM. *, *P* < 0.05. Statistical analysis in panels (C, D) was performed by the one‐way ANOVA with multiple comparisons. (E) IHC staining of hepatic Col I enhanced by DAB. (F,G) Western blotting for hepatic Col I, Col III, Col IV, and GAPDH. *Zip13 OE* (overexpression throughout the body*, Rosa‐Cre^+/^* ^−^ *Zip13OE^+/^* ^−^) mice group *VS* control (*Zip13OE ^fl/fl^*) group was compared in G. n = 6. Data are shown as the mean ± SEM. *, *P* < 0.05, for Col I; #, *P* < 0.05, for Col III; $, *P*< 0.05, for Col IV. Statistical analysis was performed by the two‐sided Student's t‐test. (H) Masson's and Sirius red staining of the liver. Scale bars, 200 µm in (B,E,H).

In the results described above, we show that, after 1 month (short‐term) of CCl_4_ treatment, ZIP13 deficiency reduces collagen formation, while, paradoxically, increasing hepato‐sensitivity toward CCl_4_. Consistently, ZIP13 overexpression (*Rosa‐Cre^+/^* ^−^ *Zip13OE ^+/^* ^−^) was associated with more fibrogenesis, and appeared to be mildly protective against CCl_4_ toxicity (Figures 2C,D; S2F,G and S5A–D). To further delineate the effect of ZIP13 overexpression (*Rosa‐Cre^+/^* ^−^ *Zip13OE ^+/^* ^−^) on liver fibrosis, which typically takes more time to fully develop, we examined these mice after 2 months (long‐term) of CCl_4_ administration. ZIP13 overexpression (*Rosa‐Cre^+/^* ^−^ *Zip13OE^+/^* ^−^) mice developed significantly firmer fibrotic livers and splenomegaly compared to WT mice (Figures 4A,B; S6A), indicating accelerated cirrhosis progression. Serum ALT and AST levels were markedly elevated in ZIP13 overexpression mice relative to WT controls after 2 months (Figure 4C,D). Histopathological analysis confirmed enhanced collagen deposition, as evidenced by increased Col I immunoreactivity (Figures 4E; S6B), western blotting (Figure 4F,G), intensified Masson's trichrome staining (Figures 4H; S6C), and elevated Sirius red positivity (Figures 4H; S6D). These findings collectively demonstrate that ZIP13 overexpression potentiates chronic hepatic fibrogenesis and cirrhosis development.

### 2.4 *Zip13 KO* in HSCs Prevents the Development of Liver Fibrosis and Cirrhosis without Exacerbating Liver Injury

**FIGURE 5:**
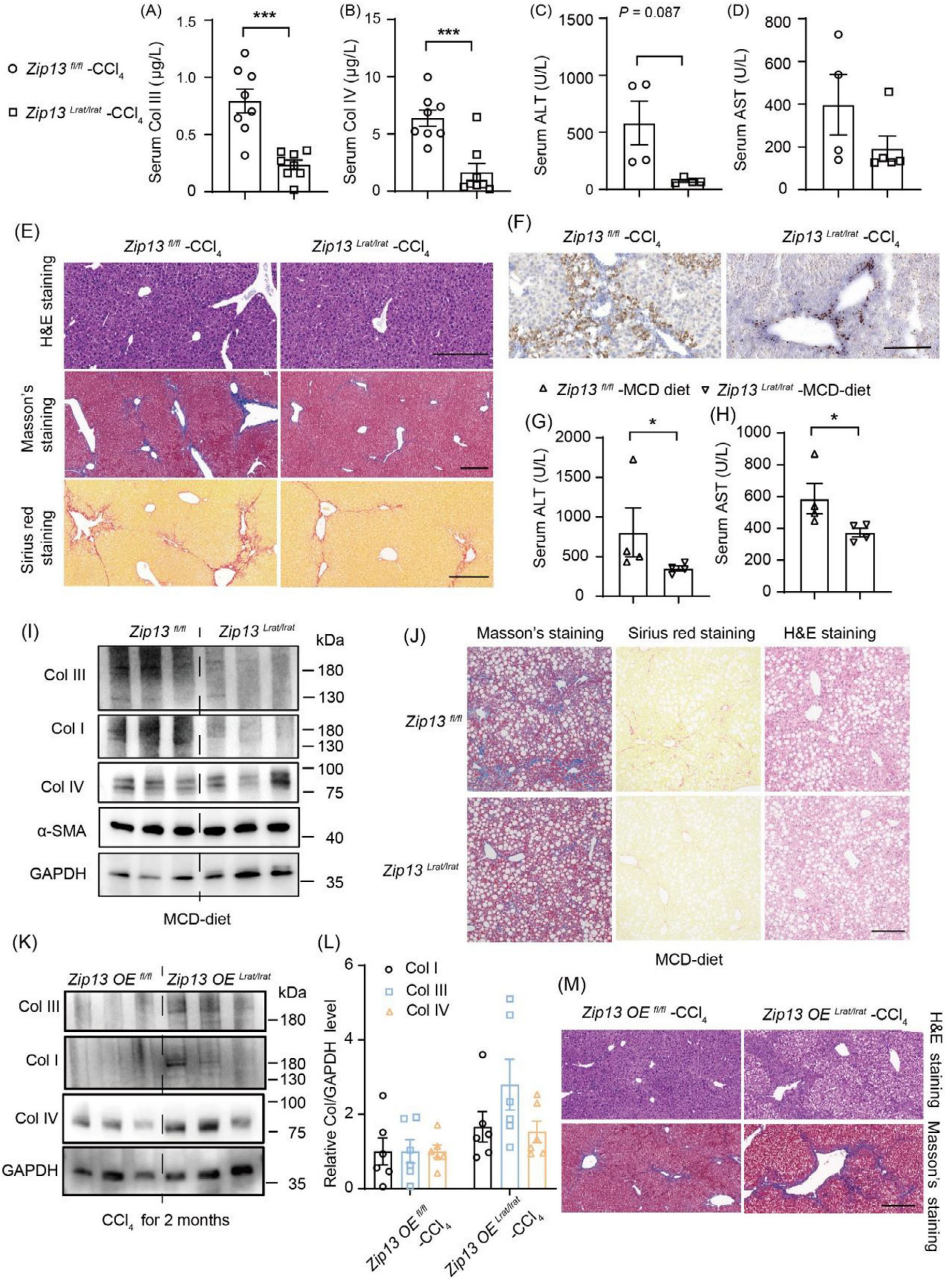
*Zip13* KO in HSCs suppressed liver fibrosis without causing liver injury. (A) Serum Col III, (B) Col IV, (C) ALT, (D) AST, (E) Hepatic H&E, Masson's and Sirius red staining, (F) IHC staining for Col I enhanced by DAB for *Zip13^Lrat/Lrat^* (*Zip13* knockout in HSCs) and *Zip13^fl/fl^* control mice after 1 month of CCl_4_ treatment. n = 8 in (A,B), n = 4 in (C,D). (G) Serum ALT, (H) serum AST, (I) Western blotting for hepatic Col I, Col III, and Col IV, (J) Masson's staining, Sirius red staining and H&E staining for liver sections from 5‐month‐old *Zip13^fl/fl^* and *Zip13^Lrat/Lrat^* mice fed with a methionine‐choline deficient (MCD)diet for 1.5 months. n = 4 in (G,H). (K,L) Western blotting for hepatic Col I, Col III, and Col IV, (M) H&E staining, and Masson's staining of the liver from the *Zip13OE^Lrat/Lrat^* (*Zip13* overexpression in HSCs) mice and their floxed control littermates (*Zip13OE^fl/fl^*) after 1 month of CCl_4_ treatment. Scale bars, 200 µm in (E,F,J,M). All data are shown as the mean±SEM. *, *P* < 0.05; ***, *P* < 0.001. Statistical analysis was performed by the two‐sided Student's t‐test.

HSCs are the primary collagen‐producing cells in the liver during liver fibrosis, responsible for most collagen synthesis and secretion. We wondered whether we could avoid ZIP13‐related hepatocyte toxicity while harnessing its involvement in collagen formation for fibrogenesis treatment by specifically targeting ZIP13 in HSCs. To this end, we used *Lrat‐Cre* mice to generate HSC‐specific *Zip13 KO* mice (*Zip13^Lrat/Lrat^*, Figure S2B,C) and confirmed expression knockdown in isolated primary HSCs (Figure S2D,E). Two‐month‐old *Zip13^Lrat/Lrat^* mice exhibited normal liver histology without evidence of injury (Figure S7A–C). Following 1 month of CCl_4_ exposure, *Zip13^Lrat/Lrat^* mice demonstrated significantly reduced collagens in the serum (Figure 5A,B), liver injury (Figure 5C–E), and hepatic collagen deposition (Figures 5E,F; S7D) compared to littermate controls (*Zip13^fl/fl^*). In addition to the CCl_4_‐induced fibrosis model, ZIP13's involvement in the fibrosis of the metabolic dysfunction‐associated steatohepatitis (MASH) model was also examined and confirmed. Lastly, we challenged mice with a methionine‐ and choline‐deficient Diet (MCD diet), and found that HSC‐specific disruption of ZIP13 could similarly prevent the progression of liver fibrosis from MASH (Figure 5G–J). Conversely, HSC‐specific *Zip13* overexpression accelerated collagen accumulation (Figure 5K–M). Overall, these results suggest that ZIP13 expressed by HSCs is a promising therapeutic target for attenuating liver fibrosis.

Col IV plays an important physiological role in maintaining vascular basement membranes and is overproduced during the early stages of liver fibrosis, altering vascular permeability. Apart from HSCs, Col IV is partially synthesized and secreted by liver sinusoidal endothelial cells (LSECs). To investigate the role of ZIP13 in LSECs during fibrosis, we prepared LSEC‐specific *Zip13* knockout mice (*Zip13^Tie−2/Tie−2^*) (Figures S2A,I and S8A,B). After 1 month of CCl_4_ treatment, *Zip13^Tie−2/Tie−2^* mice exhibited lower levels of Col IV in both the serum and liver when compared with the littermate controls (Figure S8C–I), but no change in Col III or Col I (Figure S8D–I), as well as no appreciable change in the extent of liver injury (Figure S8E,F,J). These results suggest that modulating ZIP13 expression in LSECs could alter the progression of liver fibrosis, but to a much lesser extent than when it is modulated in HSCs.

### 2.5 ZIP13 Mediates ER/Golgi Iron Homeostasis and Collagen Maturation

**FIGURE 6:**
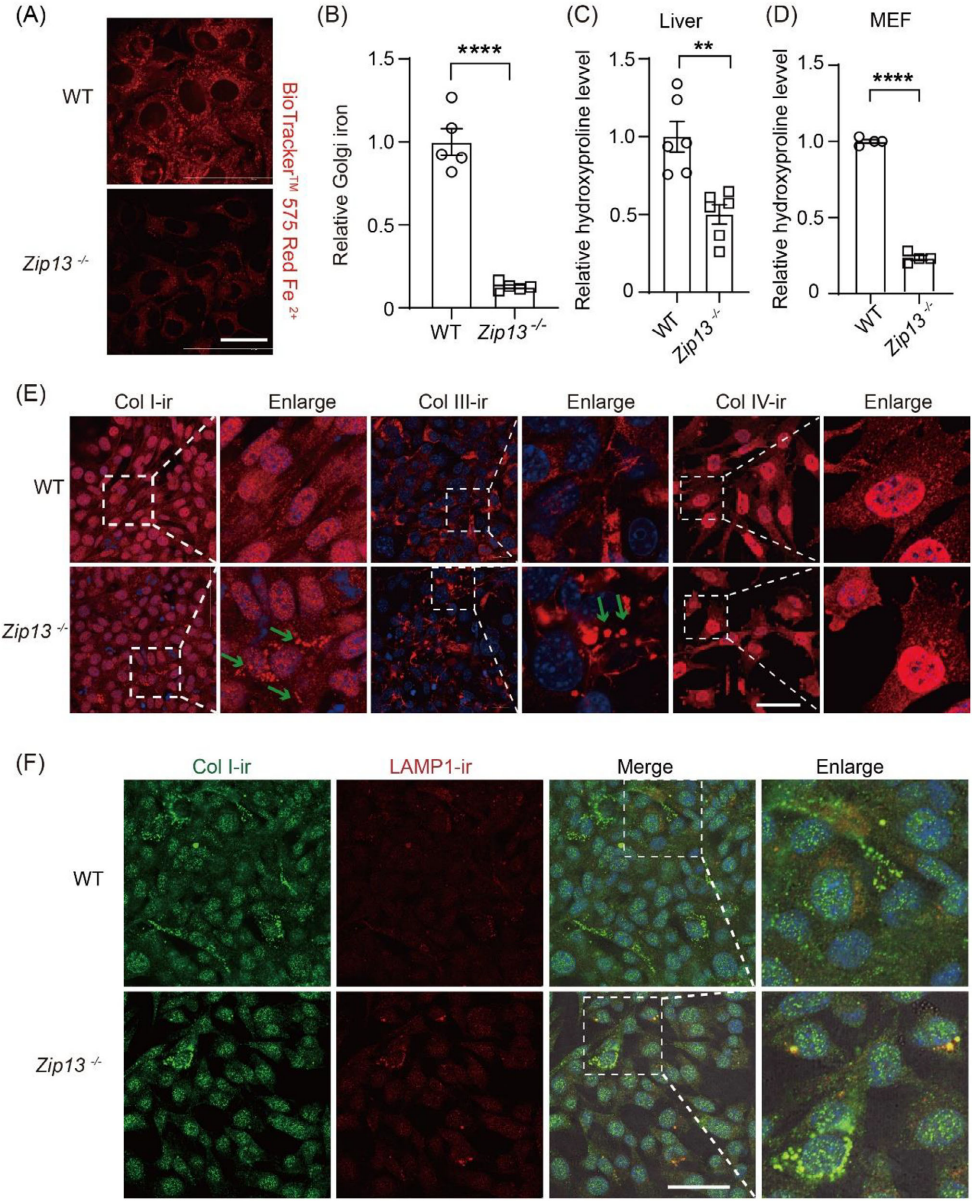
ZIP13 mediated ER/Golgi iron and intracellular collagen maturation. (A,B) Golgi iron of primary HSCs from WT and *Zip13* ^−^ *^/^* ^−^ mice, n = 5. (C,D) Relative hydroxyproline levels in the liver from WT and *Zip13* ^−^ *^/^* ^−^ mice (n = 6), and from WT and *Zip13* ^−^ *^/^* ^−^ immortalized fibroblasts (MEFs) (n = 4). (E) IF staining for Col I (red), Col III (red), and Col IV (red) in MEFs from WT and *Zip13* ^−^ *^/^* ^−^ mice. The nucleus is stained using DAPI (blue). The green arrows represent the assembled Col I fibers in the WT cells and unassembled Col I in the *Zip13* ^−^ *^/^* ^−^ cells. (F) Co‐staining for Col I (green) and LAMP‐1 (red) in MEFs. The nucleus is stained by DAPI (blue). Apparent intracellular collagen accumulation was observed in *Zip13* ^−^ *^/^* ^−^ *cells*. Scale bars: 50 µm in (A,E,F). Data are shown as the mean ± SEM. **, *P* < 0.01; ****, *P* < 0.0001. Statistical analysis was performed by the two‐sided Student's t‐test.

Lysine and proline hydroxylation is indispensable for collagen crosslinking and maturation. Our group has demonstrated that ZIP13 is the major, if not the sole, transporter responsible for transferring iron from the cytosol to the ER/Golgi and that this iron is necessary for normal collagen hydroxylation in primary embryonic MEFs [24]. To confirm that this also occurs in our HSCs, we analyzed ER/Golgi iron levels in *Zip13* ^−^ *^/^* ^−^ HSCs. ER/Golgi iron levels in *Zip13* ^−^ *^/^* ^−^ HSCs were significantly decreased (Figure 6A,B), accompanied by decreased hydroxylated proline in *Zip13* ^−^ *^/^* ^−^ livers (Figure 6C) and MEFs (Figure 6D). Col I and Col III failed to form assembled fibers intracellularly or extracellularly in *Zip13* ^−^ *^/^* ^−^ cells (Figure 6E). Interestingly, Col I co‐localized with LAMP1‐positive lysosomes, possibly representing the partial lysosomal degradation of misfolded collagen (Figure 6F). This lysosomal degradation pathway suggests that compromised collagen structures may undergo efficient clearance by either producing cells or neighboring macrophages. While untested, ZIP13 likely also impacts other HSC‐secreted collagens (e.g., IV, V, VII), given their similarity. Collectively, these findings establish ZIP13 as a key regulator of collagen assembly, cross‐linking, and maturation. Our results suggest that ZIP13 deficiency results in failed collagen secretion and enhanced collagen degradation.

### 2.6 Targeting HSC ZIP13 to Treat Liver Fibrosis

**FIGURE 7:**
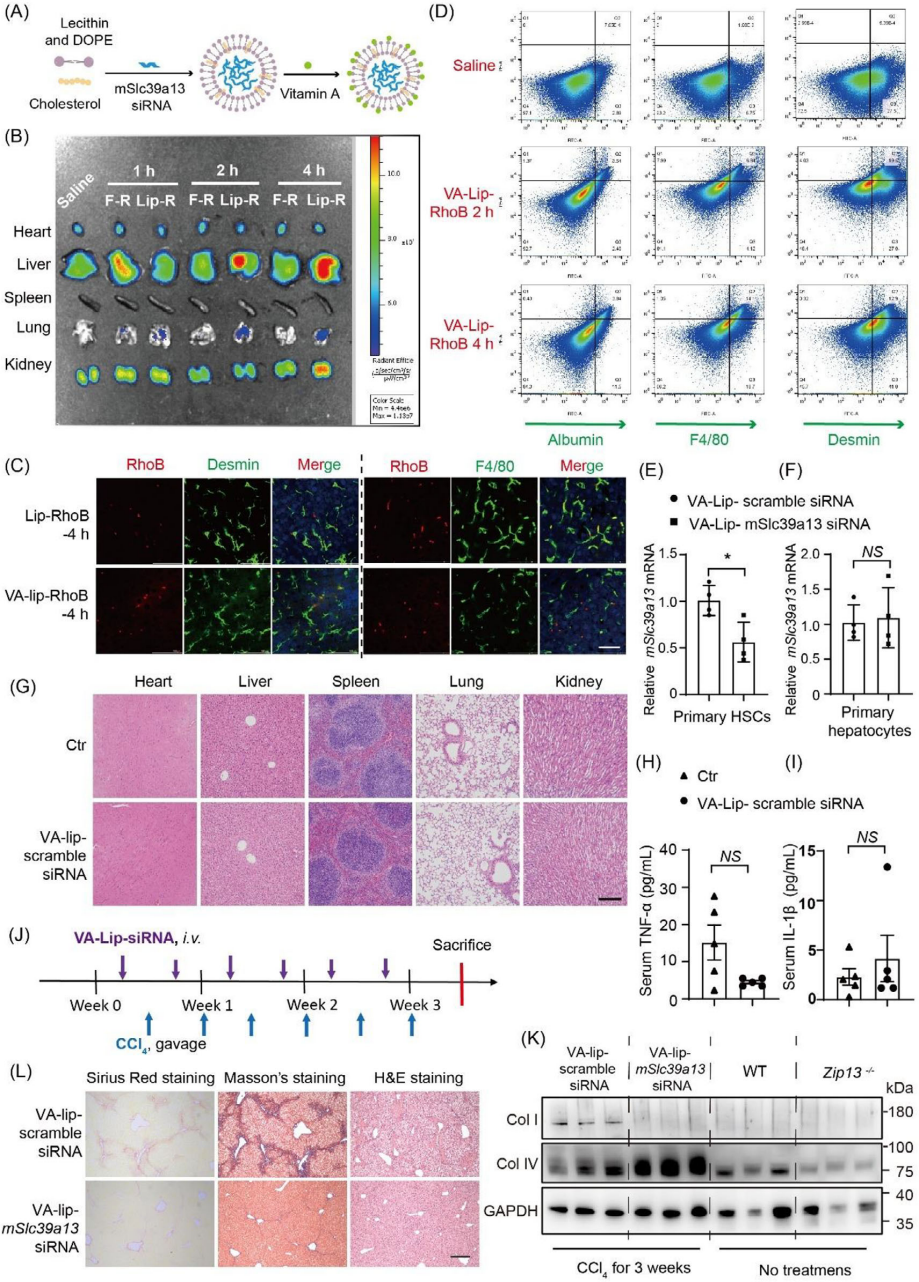
ZIP13 acted as an effective therapeutic target for the mitigation of liver fibrosis. (A) Preparation scheme for the VA‐lip *mSlc39a13* siRNAs. (B) Body distributes images by small animal imaging. F‐R means free rhodmine B, and lip‐R means VA‐lip‐rhodamine B here. (C) IF staining for desmin‐immunoreactivity (desmin‐ir) HSCs (green) and F4/80‐ir Kupffer cells (green), and (D) Flow cytometry analysis of desmin‐ir HSCs, F4/80‐ir Kupffer cells, and albumin‐ir hepatocytes from the liver of wild‐type (WT) mice intravenously injected with VA‐lip‐rhodamine B (VA‐lip R, red) for 2 h and 4 h, respectively. (E,F) Knockdown efficiency of *Slc39a13* siRNA in the primary HSCs and hepatocytes isolated from VA‐lip *mSlc39a13* siRNA injected mice, n = 4. (G) H&E staining of the heart, liver, spleen, lung and kidney, and plasma (H) TNF‐α and (I) IL‐1β levels from mice administered VA‐lip‐scramble siRNAs. n = 5. (J) Time schedule for administration of VA‐lip *mSlc39a13* siRNAs for WT mice induced by CCl_4_ gavage for 3 weeks. (K) Western blotting for Col I, Col IV, and GAPDH, (L) Sirius red staining, Masson's staining, and H&E staining for the liver from mice administered VA‐lip *mSlc39a13* siRNAs. The WT or *Zip13* ^−^ *^/^* ^−^ group with no treatments was used as negative control in (K). Scale bars, 50 µm in C, 200 µm in G and L. Data are shown as the mean ± SEM. *NS*, no significant; *, *P* < 0.05. All the statistical analysis was performed by the two‐sided Student's t‐test.

The above results suggest that ZIP13 in HSCs is a promising therapeutic target for safely attenuating liver fibrosis progression. To test this hypothesis, we employed vitamin A‐modified liposomes (VA‐Lips), previously validated for HSC‐specific delivery [10], to administer *Slc39a13* siRNA (VA‐Lip‐*mSlc39a13* siRNA, with VA‐Lip‐scramble siRNA as controls) (Figures 7A; S9A,B). Rhodamine B‐labeled VA‐Lips (Lip‐R) demonstrated preferential hepatic accumulation versus extrahepatic tissues (Figure 7B). Within liver sections, Lip‐R predominantly localized to desmin^+^ HSCs rather than albumin^+^ hepatocytes, with partial uptake by F4/80^+^ Kupffer cells due to their phagocytic function (Figures 7C,D; S9C,D). We also show that the siRNAs could escape from the lysosome (Figure S9E).

The VA‐Lip‐*mSlc39a13* siRNAs were able to knock down *Zip13* in HSCs by 50% without affecting the expression of *Zip13* in hepatocytes (Figure 7E,F). We then tested the biosafety of the lipid nanoparticles and found neither siRNA delivery system itself (Figure 7G–I) nor VA‐Lip‐*mSlc39a13* siRNAs (Figure S9F–I) caused obvious inflammatory response and organ damage. To test whether VA‐Lip‐*mSlc39a13* siRNAs could alleviate the fibrogenesis effect of CCl_4_ treatment, mice were injected intravenously with liposomes as shown in the timeline (Figure 7J). Compared to VA‐lip‐scramble siRNA controls, the VA‐lip‐*mSlc39a13* siRNA substantially inhibited collagen deposition following 1 month of CCl_4_ exposure (Figures 7K,L; S9J).

## 3 Discussion

**FIGURE 8:**
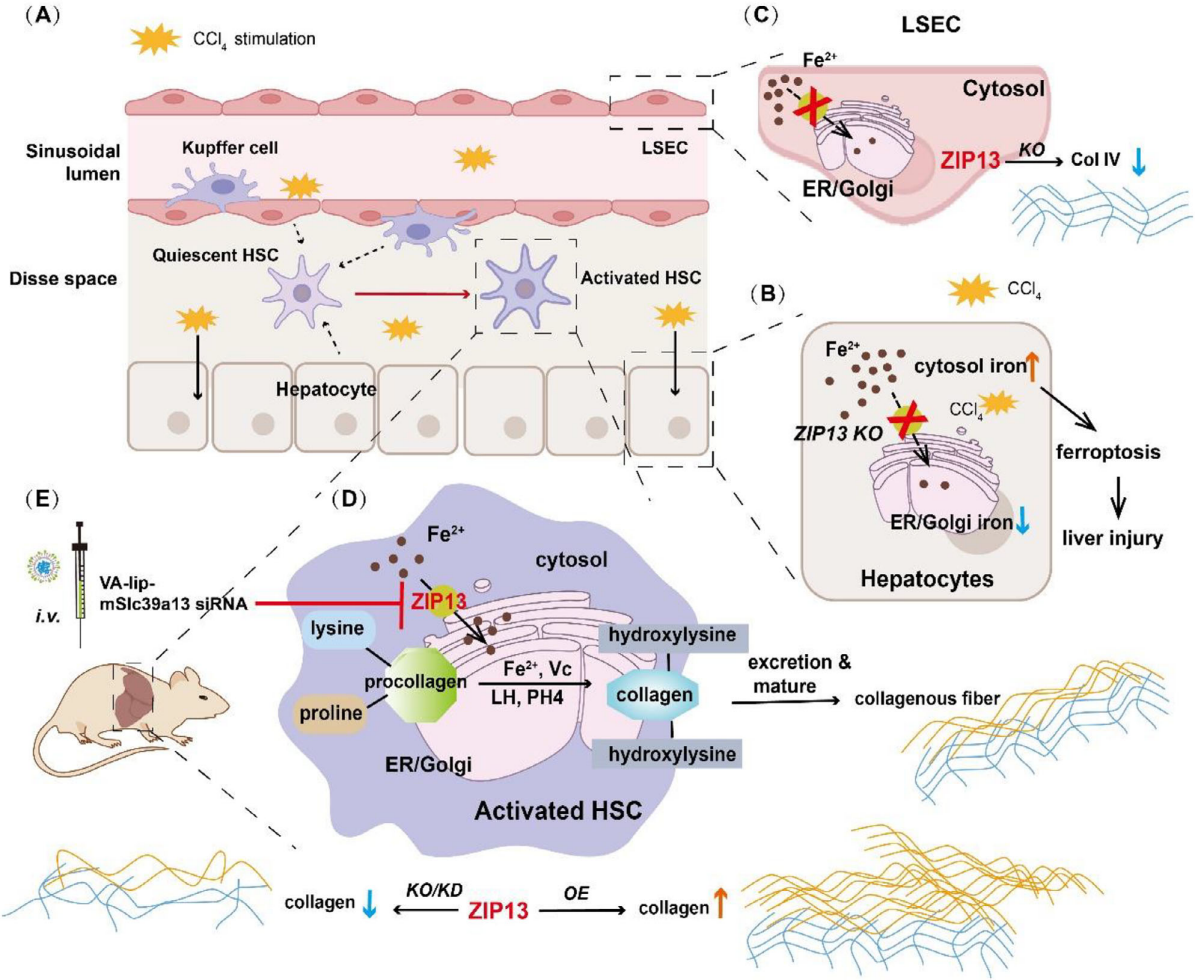
A schematic illustrating the roles of ZIP13 in distinct liver cell populations during liver fibrogenesis. (A) Following CCl_4_ stimulation, hepatocytes, liver sinusoidal endothelial cells (LSECs), and hepatic stellate cells (HSCs) are all affected. (B) In hepatocytes, the primary site of hepatic iron storage, ZIP13 deficiency induces cytosolic iron accumulation. This promotes iron‐mediated toxicity (at least partially via ferroptosis), exacerbating CCl_4_‐induced hepatic injury. (C) In LSECs, which secrete Col IV to maintain the basement membrane and extracellular matrix (ECM), ZIP13 deficiency reduces Col IV deposition within the ECM. (D,E) In activated HSCs, the principal collagen‐producing cells during liver fibrosis progression, genetic ablation or knockdown of *Zip13* effectively inhibits collagen deposition within the ECM. This suppression attenuates fibrogenesis with minimal hepatic harm.

Previous research by our group established that ZIP13 is a critical component of intracellular iron trafficking. Specifically, ZIP13 facilitates cytosolic iron translocation into the ER/Golgi secretory pathway [24, 29]. Early clinical and laboratory studies confirm that ZIP13 deficiency impairs collagen synthesis, with the primary phenotype of ZIP13 mutant patients or mice being skin and bone disorders [25]. However, its potential role in pathological contexts such as liver fibrosis remains unexplored. In this study, we propose a “ZIP13‐ER/Golgi iron‐liver fibrosis” regulatory paradigm elucidating the interplay among hepatic iron metabolism, toxicity, and fibrogenesis (Figure 8). While a number of previous studies indicate that dysregulated hepatic iron metabolism (e.g., iron overload) exacerbates the risk of liver fibrosis progression, the precise mechanisms remain inconclusive and sometimes contradictory [30, 31, 32]. In this study, we demonstrated that it is subcellular iron homeostasis, specifically ER/Golgi iron availability, rather than total hepatic iron burden, that governs fibrotic progression. This finding challenges the conventional, and somewhat oversimplified, “iron overload promotes fibrosis” axiom. Mechanistically, ZIP13 facilitates cytosolic iron translocation to the ER/Golgi, enabling proline/lysine hydroxylation in procollagen peptides. This is a prerequisite for the maturation of newly formed collagen and a key process driving pathological collagen accumulation within the hepatic extracellular matrix. Thus, our work establishes a novel conceptual ZIP13‐ER/Golgi iron‐liver fibrosis framework integrating iron trafficking, collagen biochemistry, and fibrogenesis.

Despite established regulatory networks governing HSC activation and associated signaling pathways, the complexity of liver fibrogenesis has impeded the development of clinically viable targeted therapies. Recent studies identify procollagen proline/lysine hydroxylation as a therapeutic target for fibroproliferative disorders [8, 20], exemplified by CP4H inhibitors (e.g., S4682, S0885) in preclinical fibrosis models [33]. However, systemic distribution of small molecules risks collateral disruption of physiological collagen metabolism in non‐target tissues. Targeting ZIP13 in HSCs provides a precise anti‐fibrotic strategy by directly inhibiting collagen synthesis and secretion, thereby attenuating pathological deposition. This approach offers three distinct advantages. The first is broad‐spectrum efficacy. Modulation of the ZIP13‐ER/Golgi iron axis impedes maturation/secretion of multiple collagen types through shared assembly mechanisms, surpassing the limitations of targeting individual collagen molecules. The second is cellular specificity. Exploiting collagen‐production exclusivity in aHSCs selectively disrupts fibrotic collagen deposition while preserving physiological collagen metabolism in other tissues and hepatic structures. The third advantage is pathway precision. The selective disruption of collagen‐specific post‐translational modifications (e.g., hydroxylation) avoids interference with non‐collagen functions of upstream regulators (e.g., TGF‐β/Smad‐mediated proliferation/EMT) or cytoplasmic hydroxylation processes (e.g., HIF‐α stabilization). In addition, the non‐permanent siRNA‐mediated *Zip13* knockdown provides additional therapeutic control, mitigating risks of irreversible side effects. Collectively, HSC‐targeted ZIP13 inhibition represents a translatable, tissue‐specific paradigm for anti‐hepatic fibrosis intervention.

It is noteworthy that the upregulation of Col IV expression appears to precede that of ZIP13, whereas the peak expression of Col I and Col III occurs at a later time point. This timing discrepancy suggests that elevated ZIP13 levels are not a prerequisite for Col IV expression. Here, it is critical to reiterate the mechanistic distinction: ZIP13 is not required for the expression of collagen itself, but rather for procollagen crosslinking—a post‐translational event that takes place downstream of collagen synthesis. From this perspective, the induction of ZIP13 following initial collagen expression is not physiologically inconsistent. So, what causes ZIP13 upregulation? We do not have a clear answer yet. Could the enhanced collagen synthesis itself trigger ZIP13 expression—essentially signaling to the cell that increased iron supply to the ER/Golgi is required to catalyze the maturation of newly synthesized collagen? If this is true, what signaling pathways underpin this regulatory cascade? These remain open questions to date, and we hope to explore this complex yet biologically relevant regulatory network in future studies.

Another important issue concerns the fate of non‑hydroxylated collagen (lacking proline/lysine modifications) in the ER/Golgi apparatus within ZIP13‑deficient hepatic stellate cells. These aberrant collagen molecules are likely retained in the endoplasmic reticulum, recognized by the cellular quality‑control system, and subsequently degraded via the proteasome pathway or through lysosomal clearance. Nevertheless, a fraction of such defective collagen may still be secreted extracellularly. Given that collagen lacking proline/lysine hydroxylation fails to fold properly and cannot assemble into stable fibrils, it is expected to be rapidly cleared by neighboring macrophages. However, the possibility cannot be excluded that intracellular retention of unassembled collagen may induce certain cellular stress, particularly to the ER and Golgi apparatus.

Fibrotic diseases, not only liver fibrosis, represent a class of pathological processes characterized by excessive deposition of extracellular matrix (particularly collagen) and abnormal scar formation in tissue architecture, which can affect multiple organs such as the lungs, liver, heart, and kidneys. Despite variations in the pathogenesis of fibrosis across different tissues, the post‐translational modification of most collagen types within the endoplasmic reticulum follows a conserved mechanism, involving the iron and ascorbate‐dependent hydroxylation of lysine and proline residues, catalyzed by lysyl hydroxylase and prolyl hydroxylase enzymes. The formation of hydroxyproline and hydroxylysine is essential for the correct folding of collagen into its thermostable triple‐helical conformation and subsequent maturation into cross‐linked fibers. Our results demonstrated the regulatory role of ZIP13 during liver fibrosis, and ZIP13 in the hepatic stellate cells is a potential therapeutic target for liver fibrosis. Encouragingly, we observed elevated ZIP13 levels in the bone marrow mesenchymal stromal cells of primary myelofibrosis patients compared to healthy controls from the public dataset (Figure S10) (GSE44426) [34]. Consequently, targeting ZIP13 in collagen‐secreting cells (e.g., within fibroblast‐like cells) in diverse fibrotic diseases to control aberrantly elevated collagen production may represent a promising and novel therapeutic strategy for these fibroproliferative disorders.

## 4 Experimental Section

### 4.1 Animal Studies

*Zip13^+/^* ^−^ mice on C57BL/6N background, and *Zip13^fl/fl^*, *Zip13OE^fl/fl^*, *Lrat‐cre* (C001205) on C57BL/6J, were purchased from Cyagen (Suzhou, China). Schemes of generating *Zip13* ^−^ *^/^* ^−^, *Zip13^fl/fl^* and *Zip13OE ^fl/fl^* mice were detailed in Figures 2g and S3d,e in a previous publication from our group [24]. Briefly, *Zip13* knockout (*Zip13* ^−^ *^/^* ^−^) mice were generated using the CRISPR‐Cas9 method by Cyagen (www.cyagen.com). The *Zip13* gene consists of ten exons, with the ATG start codon located in exon 2 and the TAA stop codon in exon 10. Two guide RNAs (gRNAs) were designed to target exon 2 of *Zip13*. The genotyping primer sequences are provided in the supplementary table, and representative DNA gel images confirming the *Zip13* ^−^ *^/^* ^−^ genotype are presented in Figure S2E. The company supplied *Zip13* heterozygous (*Zip13^+/^* ^−^) mice, and *Zip13* homozygous knockout (*Zip13* ^−^ *^/^* ^−^) mice along with their wild‐type (WT) littermates were subsequently obtained by intercrossing *Zip13^+/^* ^−^ mice. To generate *Zip13*‐conditional knockout mice (*Zip13^fl/fl^*), exon 2 of the *Zip13* gene was flanked by loxP sites. Cre‐mediated recombination induces the deletion of exon 2, which contains a 301‑bp coding sequence including the start codon. These mice were acquired from Cyagen. The genotyping primer sequences are provided in the supplementary table, and representative DNA gel images confirming the *Zip13^fl/fl^* genotype are shown in Figure S2A.

ZIP13‑overexpressing mice (*Zip13OE^fl/fl^*) were produced by inserting a ZIP13‑HA cassette into the mouse ROSA26 locus (Figure S2F). The targeting strategy was designed and implemented by Cyagen. A donor vector carrying a “CAG promoter–loxP–PGK‑Neo–6*SV40 pA–loxP–Kozak–mouse *Slc39a13* (*Zip13*) genomic sequence–HindIII‑HA–rBG pA” construct was co‑injected with Cas9 mRNA into fertilized mouse eggs to obtain conditionally targeted knock‑in offspring (ZIP13‑HA). *Rosa‐Cre* (B6.129‐Gt(ROSA)26Sortm1(cre/ERT2)Tyj/J, Stock No. 008463) and *Tie‐2‐Cre* (B6.Cg‐Tg(Tek‐cre)1Ywa/J, strain #:008863) were from the Jackson Laboratory. Mice with conditional knockout of *Zip13* in specific cell types were generated by intercrossing the offspring of *Zip13^fl/fl^* mice with *Lrat‐Cre* (hepatic stellate cells), *Alb‐Cre* (hepatocytes) or *Tie‐2‐Cre* (liver sinusoidal endothelial cell) mice, and their *Zip13^fl/fl^* littermates were used as the controls. The WT C57BL/6J mice used for establishing the liver fibrosis models were purchased from the Beijing Vital River Laboratory Animal Technology Co., Ltd. Upon arrival at the animal house, mice were kept under specific pathogen‐free conditions, with controlled humidity at a temperature between 20°C and 26°C, on a 12 h/12 h light/dark cycle and with free access to food and water. All the animal studies were approved by the Institutional Animal Care and Use Committee of Shenzhen Institute of Advanced Technology (SIAT‐IACUC‐220617‐HCS‐ZB‐A2162) and the Institutional Animal Care and Use Committee of the Shenzhen Brain Science Infrastructure (240923‐HCS‐ZB‐A0087) in Shenzhen, China.

### 4.2 Treatments and Analysis for Mouse Model of Liver Fibrosis

Three mouse models of liver fibrosis were used in this work: CCl_4_, acetaminophen (APAP), and the methionine‐ and choline‐deficient diet (MCD diet) treated models. For CCl_4_ treatment, 2‐month‐old wild‐type (WT) mice were gavaged with CCl_4_ for 1 or 2 months (25% v/v in corn oil, 150 µl/20 g, twice a week), and the control mice were gavaged with corn oil at a dose of 150 µl/20 g. To ascertain the rescue effect, deferiprone (DFP, 50 mg/kg) or ferrostatin‐1 (fer‐1, 5 mg/kg) were intraperitoneally injected into the mice 1 day before CCl_4_ gavage (25% v/v in corn oil, 150 µl/20 g, twice a week gavage) and continued for 1 month. For the APAP‐ induced model, 2‐month‐old mice were intraperitoneally injected (*i.p*.) with a dose of 250 mg/kg APAP every 2 days for 1 month. For the MCD diet method, 2‐month‐old mice were fed an MCD diet for 1.5 months. Four days after the last dose of CCl_4_ gavage or the last day of the MCD diet, the mice were euthanized and perfused with cold saline (0.9%). Fresh tissues were collected and immediately stored at −80°C for later Western blotting analysis and RNA isolation. Other parts of perfused tissues were fixed in 4% paraformaldehyde overnight, and the tissue sections were examined following H&E, Sirius Red, and masson‐trichrome staining by Servicebio (Wuhan, China).

### 4.3 Isolation of Hepatocytes, Hepatic Stellate Cells (HSCs), and Liver Sinusoidal Endothelial Cells (LSECs)

The procedures in this section were performed as previously described with a minor modification [30]. All cells were cultured in a humidified incubator maintained at 37°C with 5% CO_2_. The following media compositions were used for each cell type. Hepatocyte adherent medium consisted of standard M199 (Servicebio, G4620) supplemented with 5% fetal bovine serum (FBS) (TransGen Biotech, FS301‐02), 0.5% bovine serum albumin (BSA) (Beyotime, ST2254), 1% penicillin‐streptomycin (PS) (Servicebio, G4003), 100 nm dexamethasone and 100 nm insulin. Hepatocyte Suspension Medium consisted of standard M199 (Servicebio, G4620) with 10% FBS and 1% PS, supplemented with 100 nm dexamethasone and 100 nm insulin. Hepatic stellate cells (HSCs) were cultured in standard DMEM (Cytiva, SH30243.01) with 10% FBS and 1% PS.

HBSS‐EGTA buffer was prepared by mixing 50 mL of 1× HBSS (Servicebio, G4203) with 50 µL of 0.5 M EGTA (Beyotime, ST068). The collagenase mixture was prepared by dissolving 15 mg collagenase I (Servicebio, GC305013), 15 mg collagenase II (Servicebio, GC305014), and 15 mg collagenase IV (Servicebio, GC305015) in 50 mL of HBSS with CaCl_2_ (Servicebio, G4204). OptiPrep density gradient medium was prepared by diluting OptiPrep (Axis‐Shield, 1114542) to final concentrations of 11.5% or 15% (v/v) in 1× HBSS. All reagents were used according to the manufacturer's instructions and stored appropriately to maintain their efficacy.

Prior to perfusion, HBSS‐EGTA and collagenase buffers were equilibrated to 37°C using a water bath. Surgical instruments, comprising a dissection tray, 12‐gauge infusion cannula, surgical sutures, microscissors, tissue forceps, and a 20 mL syringe, were sterilized and arranged. Mice were anesthetized with isoflurane and, following loss of pedal reflexes, a midline laparotomy was performed to access the abdominal cavity. Visceral organs were carefully retracted rightward to expose the intrahepatic inferior vena cava (IVC) and portal vein.

An infusion cannula was introduced into the IVC and fixed in place with a ligature. The portal vein was immediately sectioned to establish drainage. Retrograde perfusion of HBSS‐EGTA buffer through the IVC commenced at 2 mL/min until hepatic blanching occurred (indicating blood clearance, approximately 10 min). Perfusion was then transitioned to collagenase solution at an identical flow rate until gross tissue dissociation was achieved (requiring an additional 10 min), as evidenced by the loss of hepatic structural integrity.

The excised liver was transferred to a chilled culture dish filled with HBSS. Hepatocytes were released via mechanical dissociation by rupturing the hepatic capsule with fine forceps until complete tissue disruption occurred. The resulting tissue suspension was filtered through a 70 µm nylon mesh. Hepatocytes were collected via differential centrifugation (50 ×g, 3 min). The nonparenchymal cells (NPCs) underwent centrifugation (900 ×g, 8 min) for pelleting. After discarding the supernatant, the pellet was resuspended in 5 mL of 15% OptiPrep. This suspension was layered onto a density gradient composed of 5 mL of 11.5% OptiPrep overlaid by 2 mL of HBSS, followed by centrifugation (2000 ×g, 20 min, 4°C) with brake disengaged. The cells between the 11.5% OptiPrep and HBSS was carefully collected, washed in HBSS, and centrifuged (800 ×g, 10 min). Two additional wash cycles were performed to eliminate residual OptiPrep and concentrate the cells. The final pellet was resuspended in HSC‐specific DMEM medium for subsequent plating and analysis. The attached hepatocytes and HSCs were fixed, permeabilized, and then characterized by immunofluorescence staining.

Liver sinusoidal endothelial cells (LSECs) were isolated using a commercial LSEC Isolation Kit (Immocell, IMP‐MK004). Mice were anesthetized and fixed in a supine position, then disinfection of the abdominal skin with 75% ethanol. The abdominal cavity was opened to expose the liver. A perfusion needle was inserted into the inferior vena cava and secured, followed by an incision in the portal vein. The liver was perfused sequentially via a peristaltic pump, first with Solution I (5 mL/min, 3 min) and then with Solution II (containing collagenase, 3 mL/min, approximately 5 min), until complete tissue digestion was observed. The liver was then excised, rinsed with wash buffer, and transferred into 10 mL of digestive enzyme solution. The liver capsule was carefully torn with forceps, and the tissue was incubated at 37°C for 20 min. Digestion was terminated by adding 40 mL of stop solution, and the resulting cell suspension was filtered through a 70 µm strainer. The filtrate was centrifuged (300 ×g, 10 min, 4°C), and the supernatant was discarded to remove light non‐parenchymal cells. The pellet was resuspended in wash buffer and subjected to low‐speed centrifugation (50 × g, 3 min, 4°C) to separate hepatocytes. The hepatocyte‐enriched pellet was discarded, while the supernatant was recentrifuged (50 g, 3 min, 4°C) to remove residual hepatocytes. The resulting supernatant was then centrifuged again (300 ×g, 10 min, 4°C) to collect non‐parenchymal cells.

The pellet was resuspended in 2 mL of wash buffer, carefully layered onto 20% LSEC purification medium, and centrifuged (600 ×g, 5 min, 4°C). The resulting cell pellet was resuspended in sorting buffer and adjusted to a concentration of approximately 1 × 10^8^ cells mL^−^^1^. For magnetic labeling, 100 µL of the cell suspension was mixed with 2 µL of the specified reagent and incubated (4°C, 15 min). The mixture was then diluted with 1 mL of sorting buffer and centrifuged (500 ×g, 5 min, 4°C). The pellet was resuspended in 100 µL of sorting buffer, followed by the addition of 10 µL of sorting magnetic beads and incubation (4°C, 10 min). After adding 1 mL of sorting buffer and centrifugation (500 ×g, 5 min, 4°C), the pellet was resuspended in 1 mL of sorting buffer and applied to a pre‐wetted sorting column in the magnetic hopper grate, ensuring no air bubbles were introduced. The column was washed three to four times with 1 mL of sorting buffer each time, and unbound cells were collected in the flow‐through. The column was then removed from the magnetic stand, placed in a new collection tube, and 1 mL of LSEC culture medium was added. Bound cells were eluted using the plunger. The eluted LSECs were adjusted to the desired cell density and plated for subsequent culture.

### 4.4 Western Blotting

Tissue and isolated cells were homogenized in RIPA buffer (Solarbio, R0010) supplemented with protease inhibitor cocktail (Roche, 4693159001) using a bead homogenizer (SAIERTE). Post‐centrifugation (12,000 ×g, 15 min, 4°C), supernatants were collected for protein analysis. Protein was quantification using a BCA assay kit (Beyotime, P0010), followed by separation on 10% SDS‐polyacrylamide gels. Electrophoretically resolved proteins were transferred to 0.45 µm PVDF membranes, and the membranes were blocked for 1 h in blocking buffer and then incubated overnight at 4°C with primary antibodies (anti‐Col I, Proteintech, 14695‐1‐AP; anti‐Col III, proteintech, 22734‐1‐AP; anti‐Col IV, proteintech, 15191‐1‐AP; anti‐αSMA, proteintech, 14395‐1‐AP; anti‐GAPDH, Servicebio, GB15004‐100) in the same buffer. The blots were then washed in TBST (TBS buffer containing 0.1% Tween‐20), incubated for 1 h with secondary antibodies (Goat Anti‐Rabbit IgG (H&L)‐HRP (BE0101, EASYBIO); Goat Anti‐Mouse IgG (H&L)‐HRP (BE0102, EASYBIO)), and detected using the Tanon 5200 imaging system (Tanon Science & Technology, China).

### 4.5 Isolation for RNA and Q‐PCR

Total RNA was extracted from cells and tissues using a commercial isolation kit (BioFlux, BSC52M1). Reverse transcription was performed using the TransGe Biotech system (AE311). Quantitative real‐time PCR analysis employed 1 µg cDNA template amplified with TransStart Top Green qPCR SuperMix (TransGen Biotech, AQ131) using gene‐specific primers. Relative gene expression levels were normalized to mGAPDH or 18S ribosomal RNA as endogenous controls.

### 4.6 ROS Measurement

Liver cells were isolated as described above. Following washing with serum‐free medium, 1 × 10^6^ cells were incubated with 10 mmol/L DCFH‐DA (MCE, Monmouth Junction, NJ, USA) at 37°C for 30 min in the dark to measure cytosolic ROS levels. The cells were then washed twice with serum‐free medium, and fluorescence intensity was quantified using a SpectraMax i3x plate reader (Molecular Devices) at excitation and emission wavelengths of 488 and 530 nm, respectively.

### 4.7 MDA Measurement

MDA levels were measured using the Lipid Peroxidation MDA Assay Kit (Beyotime, S0131S). Liver samples were homogenized with RIPA lysis buffer at 4°C and were quantified using a BCA assay kit. The liver samples were then analyzed in accordance with the manufacturer's instructions.

### 4.8 SOD Activity Measurement

SOD activities in the liver of mice induced by CCl_4_ were measured using the “Total Superoxide Dismutase Assay Kit ” (Beyotime, S0101S) and following the manufacturer's instructions.

### 4.9 GPX4 Activity Measurement

GPX4 activity in mouse liver tissue was quantified using a Glutathione Peroxidase 4 (GPX4) Activity Assay Kit (Elabscience, E‐BC‐K883‐M), according to the manufacturer's protocol. The assay principle is as follows: the oxidation product generated from the GPX4‐catalyzed reaction depletes the reducing agent, which has a characteristic absorbance peak at 340 nm. GPX4‐specific activity was determined by measuring total enzyme activity and non‐specific enzyme activity (in the presence of a GPX4 inhibitor) and calculating the difference. The final GPX4 activity values were normalized to the total protein concentration as determined by a BCA protein assay kit (Beyotime, P0010).

### 4.10 ELISA

Elisa for serum HA (Dogesce, DG30585M), LN (Dogesce, DG30283M), PC III (MREDA, M150561), and Col IV (Dogesce, DG30307M) were measured following the manufacturers’ instructions.

### 4.11 Synthesis and Characterization of VA‐lip mSlc39a13 siRNAs

VA‐lip mSlc39a13 siRNAs were synthesized per established protocols with modifications [10]. Briefly, cationic lipid DC‐6‐14, cholesterol, and DOPE (4:3:3 molar ratio) were dissolved in dichloromethane (DCM). Rotary evaporation under vacuum yielded a lipid film in a round‐bottom flask. After adding nuclease‐free water, the lipid film was hydrated with vortex mixing followed by water bath sonication. Liposome formation was achieved by extrusion through polycarbonate membranes using a liposome preparation device (Avanti, 610020). Subsequent centrifugation (14 000 ×g, 20 min, 4°C) with distilled water removed unencapsulated materials. Vitamin A conjugation and siRNA encapsulation followed published methodology [10]. Specifically, 200 nmol retinol (Aladdin, V820461) in DMSO was mixed with liposome suspensions (100 nmol DC‐6‐14 equivalent) by vortexing (1.5 mL tube, 25°C). For VA‐coupled liposomes carrying mSlc39a13 siRNAs (VA‐lip‐mSlc39a13 siRNAs), the mSlc39a13 siRNA solution (580 pmol/mL in nuclease‐free water) was incorporated into retinol‐coupled liposomes by stirring (25°C) for 10–20 min, while maintaining siRNA:DC‐6‐14 at 1:11.5 (mol/mol) and siRNA:liposome at 1:1 (wt/wt). Free components were then removed by centrifugation (14 000 × g, 20 min, 4°C).

The size distribution and surface charge of VA‐lip‐siRNAs in distilled water were examined using a ZetaSizer Nano series Nano‐ZS system (Malvern Instruments Ltd., Malvern, UK) equipped with a He‐Ne laser beam at a wavelength of 633 nm and a fixed scattering angle of 90°, at 25°C. For morphology characterization, the VA‐lip‐siRNAs were negatively stained with 2% uranyl acetate solution and deposited onto a carbon‐coated copper grid. Images were then acquired using transmission electron microscopy (TEM; JEM‐200CX; Jeol Ltd., Japan).

### 4.12 Bio‐Distribution of the VA‐lip‐RhoB

To track the biodistribution of VA‐liposomes, rhodamine B (RhoB) was encapsulated as a fluorescent tracer. RhoB‐labeled VA‐liposomes (VA‐lip RhoB) were administered via lateral tail vein injection to C57BL/6J mice. Animals were euthanized at 1, 2, and 4‐h post‐injection intervals. Major organs (heart, liver, spleen, lungs, kidneys) were excised and subjected to ex vivo imaging using a multispectral small animal imaging system (CALIPER IVIS).

Male C57BL/6J mice (2‐month‐old) were euthanized at 2 or 4 h post‐intravenous administration of lip‐RhoB or VA‐lip‐RhoB, respectively. Hepatic perfusion was performed according to the established protocol for hepatocyte and hepatic stellate cell (HSC) isolation. The resulting cell suspension was centrifuged (1000 rpm, 10 min), followed by the addition of red blood cells lysis buffer (Beyotime, C3702). The isolated hepatic cells were resuspended in HBSS and equally divided into three aliquots for immunophenotyping. All the cells were permeabilized with Perm Buffer I (BioLegend) according to manufacturer specifications. Then the cell populations were identified through the following sequential staining:

1) Primary antibody incubation (room temperature, 1 h):
‐ Hepatocytes: anti‐albumin (Proteintech, 16475‐1‐AP)
‐ Kupffer cells: anti‐F4/80 (Abcam, ab300421)
‐ HSCs: anti‐desmin (Proteintech, 16520‐1‐AP)
2) HBSS washes (×2)
3) Secondary antibody (room temperature, 1 h):
‐ anti‐Rabbit Alexa Fluor 488‐conjugated IgG (Invitrogen, A‐21206)
4) HBSS washes (×2).

### 4.13 Intracellular Trafficking of VA‐lip‐siRNA‐FAM in Hepatic Stellate Cells (HSCs)

Primary HSCs were seeded on glass‐bottom confocal dishes (Cellvis, D29‐20‐1‐N) at 70%–80% confluency. For uptake analysis, cells were exposed to VA‐lip‐siRNA‐FAM complexes for 1 or 4 h. Following treatment, fixation was performed with 1% paraformaldehyde (10 min), and the cells washed twice with PBS. The lysosomal compartment was visualized using LysoTracker Red (Beyotime Biotechnology, C1046) as per the manufacturer's protocol. Nuclear counterstaining employed DAPI (Beyotime, C1002). Fluorescence imaging was conducted on a Leica TCS SP5 confocal system.

### 4.14 Immunohistochemical and Immunofluorescent Staining

All mouse livers from VA‐lip siRNA‐treated groups underwent vascular perfusion fixation with 4% paraformaldehyde followed by a 16‐h immersion fixation. After cryoprotection in 30% sucrose, serial coronal sections (30 µm) were prepared using a cryostat. From the sections in each liver, we selected 3 to 4 sections at the same region for the detection of Col I, Col III, Col IV, and α‐SMA. The sections were incubated with primary antibody (the same antibodies as western blotting) at 4°C overnight. For immunohistochemical staining, the sections were rinsed and incubated with a biotinylated secondary antibody and SRABC‐HRP complex (P0612, P0615, Beyotime). The reaction product was visualized by incubation in 0.5% diaminobenzidine (DAB, D5905‐50TAB, sigma) and 0.01% hydrogen peroxide. The cell nuclei were visualized using hematoxylin. After washing, the sections were mounted, ethanol dehydrated, xylene cleared, and covered with adhesive coverslips for microscope slides (188105, CITOTEST). Images were captured using light microscopy (ECLIPSE TI2‐E, Nikon). For immunofluorescence staining, tissue sections were processed identically to the immunohistochemistry protocol until the step preceding secondary antibody incubation. Following primary antibody incubation and washes, sections were incubated with a fluorescent dye‐conjugated secondary antibody (Donkey Anti‐Rabbit IgG (H+L), Alexa Fluor 594, catalog #A21207, Thermo Fisher Scientific). Nuclei were subsequently counterstained with 4',6‐diamidino‐2‐phenylindole (DAPI). After final rinses, sections were air‐dried and mounted with anti‐fade mounting medium under adhesive coverslips (catalog #188105, CITOTEST). Fluorescent images were acquired using a Nikon AX confocal laser scanning microscope.

For the detection of VA‐lip RhoB in the liver, one group of sections was used for immunostaining of F4/80 (to visualize Kupffer cells), and the other group of sections was used for immunostaining of Desmin (to visualize HSCs). The sections were incubated at 4°C overnight with the F4/80 primary antibody (Abcam, ab300421) or the Desmin primary antibody (Proteintech, 16520‐1‐AP).

### 4.15 Bio‐Safety Evaluation

Serum and tissue samples from the mice treated with VA‐lip scramble siRNAs and VA‐lip mSlc39a13 siRNAs described above were examined. Serum levels of the pro‐inflammatory cytokine interleukin‐1β(IL‐1β) were determined by enzyme‐linked immunosorbent assay and tissue sections were examined following H&E staining by Servicebio company.

### 4.16 Statistical Analysis

All results are presented as means ± SEM. Two‐tailed Student's t‐test, one‐way ANOVA or two‐way ANOVA followed by post hoc analysis was used to test for differences between experimental groups when the sample followed a normal distribution. Groups were considered significantly different when *P* < 0.05. Statistics analyses were generally performed using the GraphPad Prism 7 software. Differential gene expression analysis in Figure 1F,G was performed using the R software (version 4.4.1) with the limma​ package (version 3.62.0).

## Author Contributions

S.G. and B.Z. designed the experiments. S.G. and Y.W. performed most of the experiments, S.G. did the analysis. S.G., Y.Z., Y.W., and B.L. performed the cellular and animal experiments together. B.Z., D.M.F., and S.G. conceived the project and wrote the manuscript.

## Funding

This study was supported by Shenzhen Medical Research Fund (B2402001), Guangdong Provincial Key Laboratory of Synthetic Genomics (2023B1212060054) and China Postdoctoral Science Foundation (S.G., 2022M723298).

## Conflicts of Interest

The authors declare no conflicts of interest.

## Supporting information




**Supporting File**: advs74198‐sup‐0001‐SuppMat.docx.

## Data Availability

The data that support the findings of this study are available from the corresponding author upon reasonable request.
