# Supplementary material for: The Regulatory Role of Iron Transporter SLC39A13 in Liver Fibrosis
Source: Adv Sci (Weinh). 2026 Feb 4;13(20):e16446. doi: 10.1002/advs.202516446 (PMC13067865; doi:10.1002/advs.202516446)
Supplement: Supplementary file 1 — Supporting File: advs74198‐sup‐0001‐SuppMat.docx. [file ADVS-13-e16446-s001.docx]

**The Regulatory Role of Iron Transporter SLC39A13 in Liver Fibrosis**

Shanshan Guo^1,2,#^, Yalin Wang^1,2,#^, Binyu Lu^1^, Yu Zhang^1,2^, David M. Frazer^3^, Bing Zhou^1,*^

^1^ Faculty of Synthetic Biology, Shenzhen University of Advanced Technology, Shenzhen 518107, China

^2^ Key Laboratory of Quantitative Synthetic Biology, Shenzhen Institute of Synthetic Biology, Shenzhen Institutes of Advanced Technology, Chinese Academy of Sciences, Shenzhen 518055, China

^3^ Molecular Nutrition Laboratory, QIMR Berghofer, Herston 4006 Australia

^#^ These authors contributed equally to this study.

* Corresponding author: zhoubing@suat-sz.edu.cn

**Materials and methods**

**Primers for genotyping:**

| **Animals** | **Forward primer (5’-3’)** | **Reverse primer (5’-3’)** |
| --- | --- | --- |
| *Zip13^-/-^* | ACATAGGCTGGAGCCAGCTCA | CAAGGTGACACTGGACTCCATGCT |
| *Zip13^fl/fl^* | AGATTCCAGACATAGGCTTGTAGC | AGTCAACCAAGCCAGAAGTTCAAT |
| ZIP13 OE-control | CACTTGCTCTCCCAAAGTCGCTC | ATACTCCGAGGCGGATCACAA |
| ZIP13 OE-transgenic | GTCTCCCCTCTCCCAACAGGCAC | CTTTATTAGCCAGAAGTCAGATGC |
| Rosa-Cre- control | CTGGCTTCTGAGGACCG | CCGAAAATCTGTGGGAAGTC |
| Rosa-Cre- transgenic | CGTGATCTGCAACTCCAGTC | AGGCAAATTTTGGTGTACGG |
| Lrat-Cre- control | CGGGTGGACACAGAACAATCG | AAGACTTTGTTTGGCTTACATGGC |
| Lrat-Cre- transgenic | CGGGTGGACACAGAACAATCG | ACCGACGATGAAGCATGTTTAGCTG |
| Alb-Cre-control | TGCAAACATCACATGCACAC | TTGGCCCCTTACCATAACTG |
| Alb-Cre- transgenic | GAAGCAGAAGCTTAGGAAGATGG | TTGGCCCCTTACCATAACTG |
| Tie-2 Cre- transgenic | CTCCTTGCCGCCAACTTGTA | CGCATAACCAGTGAAACAGCATTGC |

**Primers for Q-PCR**

| **Gene Name** | **Forward primer (5’-3’)** | **Reverse primer (5’-3’)** |
| --- | --- | --- |
| *mSlc39a13* | CCGCAGCTGGATGGCTG | CCCCAGCCCTTTCCAAGAG |
| *mGAPDH* | TGTGTCCGTCGTGGATCTGA | CCTGCTTCACCACCTTCTTGAT |
| *18S* | TTCTGGCCAACGGTCTAGACAAC | CCAGTGGTCTTGGTGTGCTGA |

**Sequence of siRNAs**

| **siRNA Name** | **Forward primer (5’-3’)** | **Reverse primer (5’-3’)** |
| --- | --- | --- |
| mSlc39a13-1232 | CUGCUUUCAUUGUUUGUUGAATT | UUCAACAAACAAUGAAAGCAGTT |
| mSlc39a13-1145 | GUGUUACCUGACCUCUUGGAATT | UUCCAAGAGGUCAGGUAACACTT |
| mSlc39a13-306 | GUCGCCUGGAUAAUAAAGAAATT | UUUCUUUAUUAUCCAGGCGACTT |
| scramble siRNA | UUCUCCGAACGUGUCACGUTT | ACGUGACACGUUCGGAGAATT |

**Supplementary figures and legends**


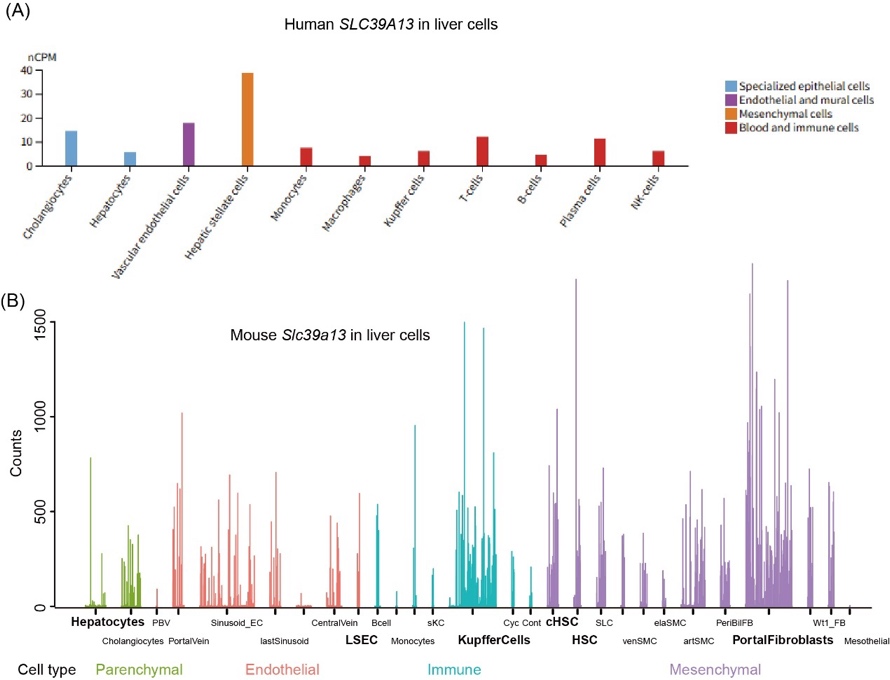


**Figure S1. RNA levels of *SLC39A13/Slc39a13* in hepatic cells from human and mouse.
(A)** Expression of *SLC39A13* (*ZIP13*) RNA in different human liver cell types. Data were obtained from the HUMAN PROTEIN ATLAS (<https://www.proteinatlas.org/ENSG00000165915-SLC39A13/single+cell/liver>). **(B)** Expression of *Slc39a13* mRNA in different mouse liver cell types. Data were derived from a public database (<https://muhldatahub.org/Publications/LiverScRNAseq/database.html>) provided by Pietilä et al. (Pietilä R, et al. *EMBO Rep*. 2025;26(21):5326–5359).

**
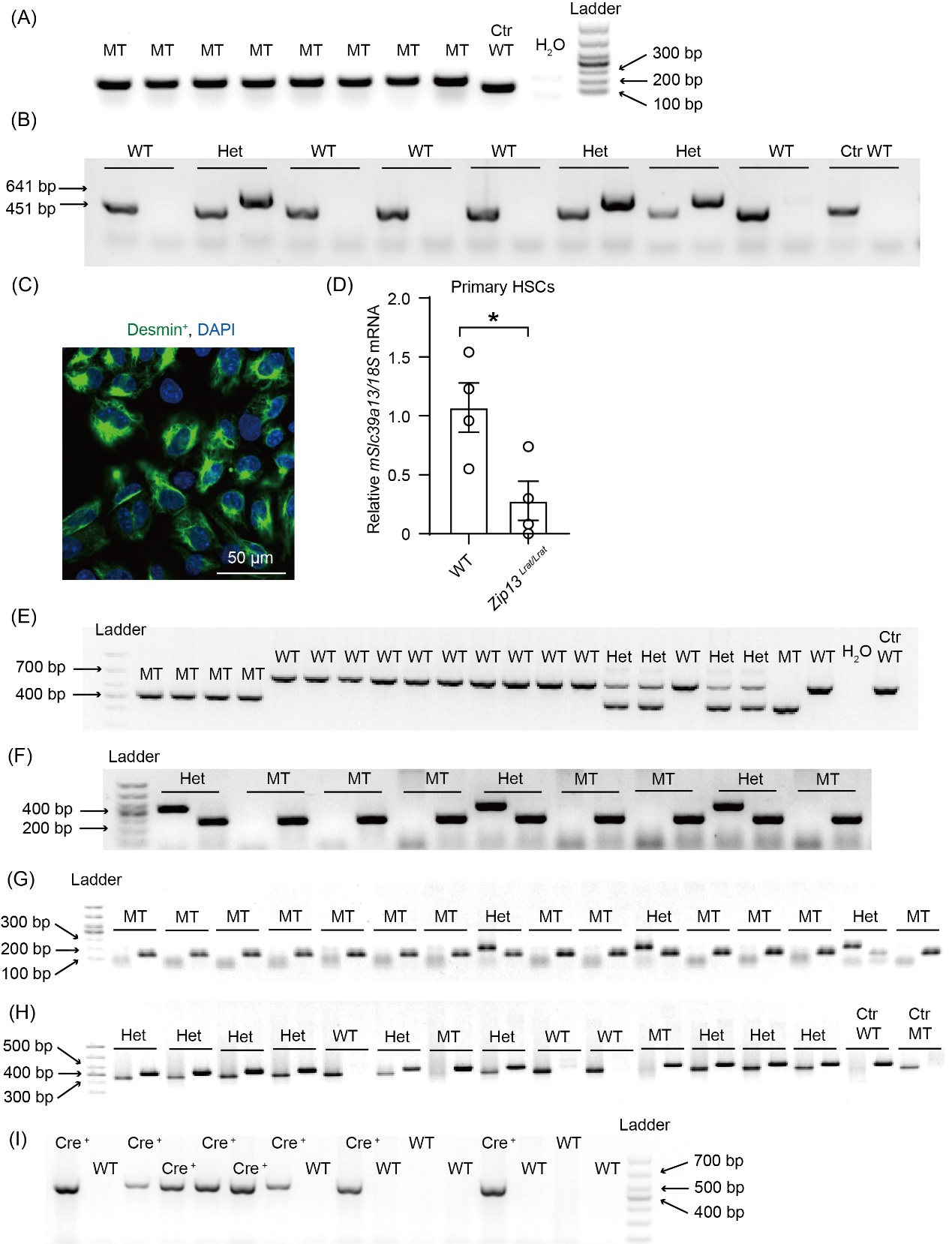
**

**Figure S2.** **Genotyping and knockout efficiency detection for the various mouse strains**. **(A)** Genotyping results for *Zip13^fl/fl^* mice (details in supplementary methods). Product size: wildtype (WT), 165 bp; targeted allele (MT): 232 bp; heterozygote (Het),165 and 232 bp. **(B)** Genotyping results for *Lrat-Cre* mice. Product size: wildtype (WT), 451 bp; targeted allele with Cre (MT): 641 bp; heterozygote (Het), 451 and 641 bp. **(C)** Immunofluorescent staining for desmin (green) in primary HSCs from the liver of mice (checking purity of the isolated cells). **(D)** Knock out efficiency of *Zip13* in hepatic stellate cells (HSCs) from the liver of *Zip13^Lrat/Lrat^* (*Zip13* knockout in HSCs) mice with WT as controls. **(E)** Genotyping results for *Zip13^-/-^* mice. Product size: wildtype (WT), 663 bp; targeted allele (MT): 418 bp; heterozygote (Het), 663 and 418 bp. **(F)** Genotyping results for *Zip13 OE^fl/fl^* mice. Product size: wildtype (WT), 453 bp; targeted allele (MT): 291 bp; heterozygote (Het), 453 and 291 bp. **(G)** Genotyping results for *Rosa-Cre* mice. Product size: wildtype (WT), 198 bp; targeted allele with Cre (MT): 150 bp; heterozygote (Het), 198 and 150 bp. **(H)** Genotyping results for *Alb-Cre* mice. Product size: wildtype (WT), 351 bp; targeted allele with Cre (MT): 390 bp; heterozygote (Het), 351 and 3390 bp. **(I)** Genotyping results for *Tie-2-Cre* mice. Product size: wildtype (WT), no bands; targeted allele with Cre (MT): about 500 bp. All the details for primers are provided in supplementary tables.


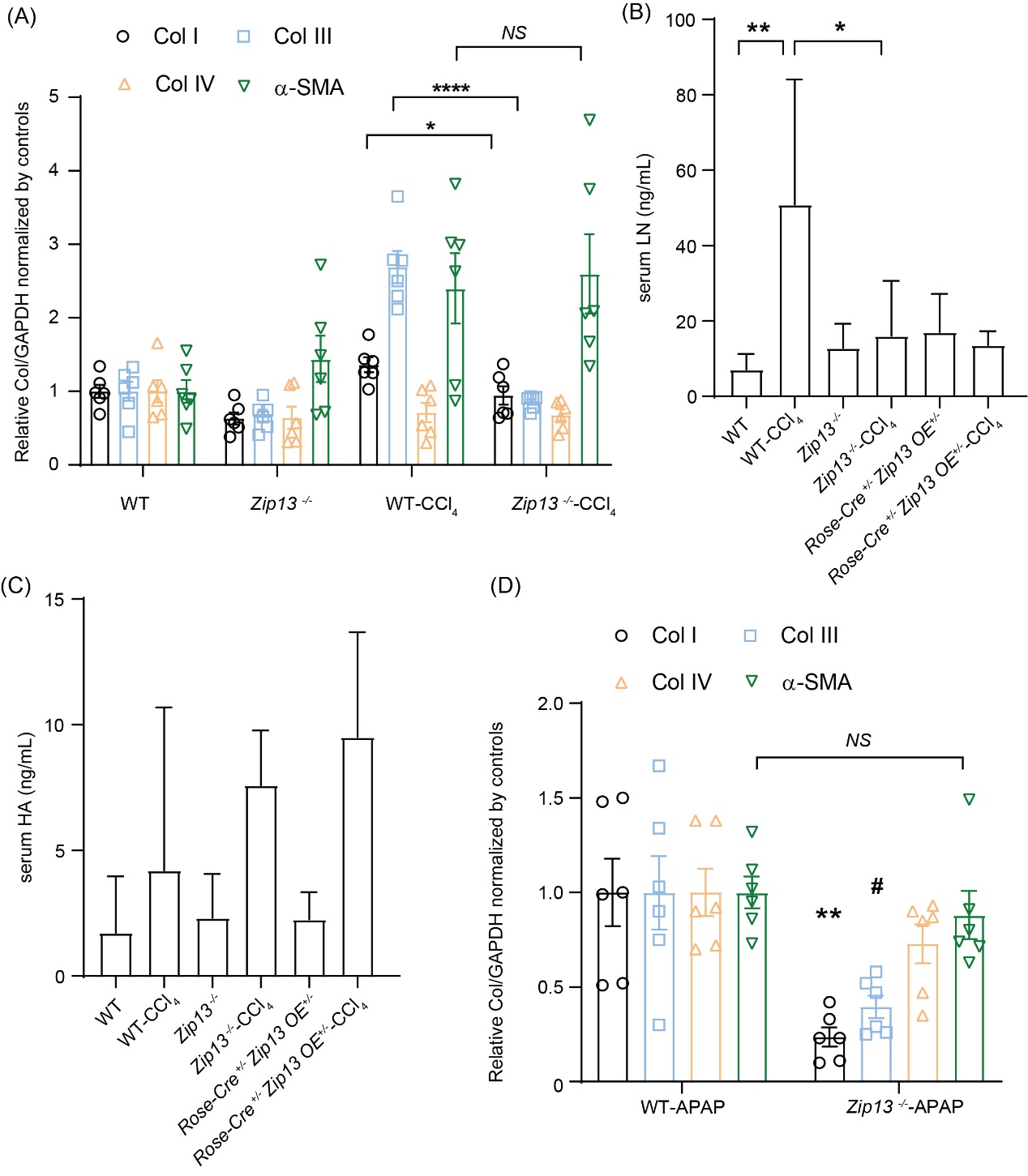


**Figure S3. ZIP13 deficiency suppressed progression of liver fibrosis in mice. (A)** Quantitative analysis of western blotting for hepatic Col I, Col III, Col IV, α-SMA and GAPDH in *Zip13^-/-^* and WT mice administrated by CCl_4_ for 1 month, n=6. Serum **(B)** laminin (LN) and **(C)** hyaluronic acid (HA) from WT, *Zip13^-/-^* and *Rosa-Cre^+/-^ ZIP13OE^+/-^*mice either untreated or treated with CCl_4_ for 1 or 2 months. n=4-6. **(D)** Quantitative analysis of western blotting results for hepatic Col I, Col III, Col IV, α-SMA and GAPDH in *Zip13^-/-^* and WT mice administrated with acetaminophen (APAP, 250 mg/kg, every two days), n=6. *Zip13^-/-^* mice group *VS* control (WT) group was compared. *NS*, no significant; **, *P* < 0.01, for Col I; #, *P* < 0.05, for Col III. Data in panels A-D are shown as the mean±SEM. In A-C, *, *P* < 0.05; **, *P* < 0.01. Statistical analysis in panels A and D was performed separately for Col I, Col III, Col IV and α-SMA by the two-sided Student’s t-test, in panels B and C by one-way ANOVA with multiple comparisons.


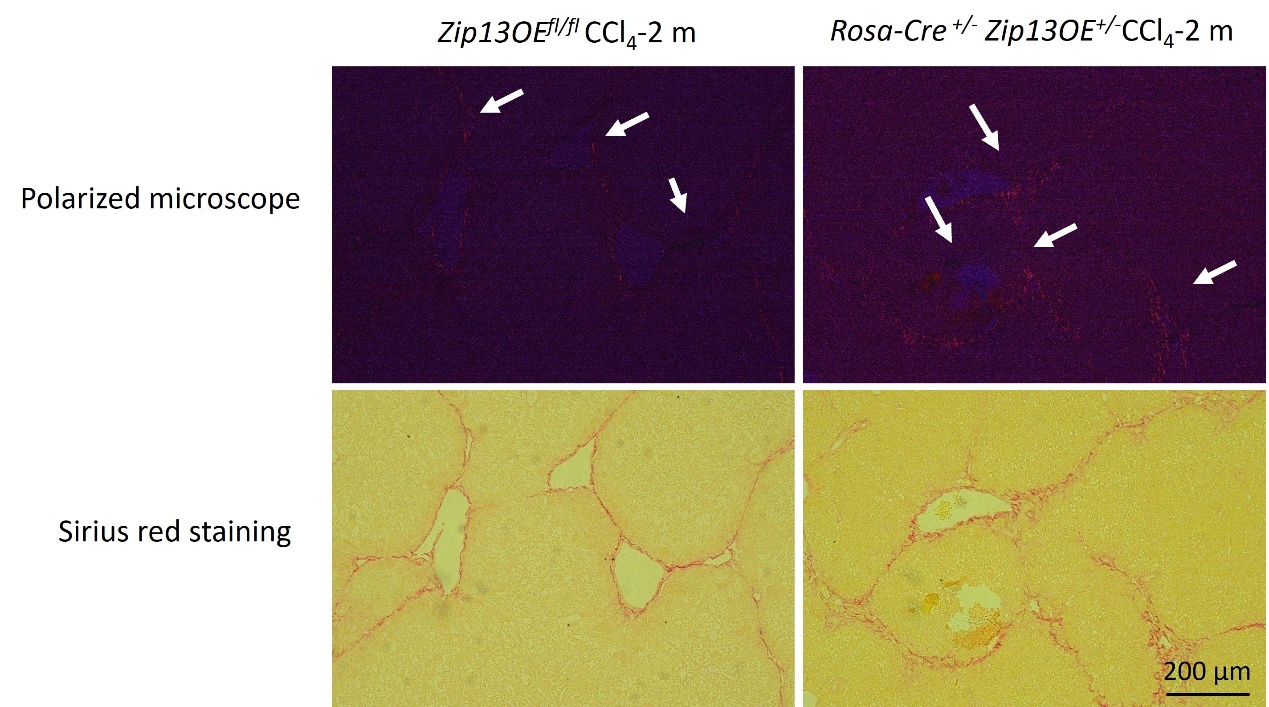


**Figure S4. The** **Sirius red staining and their polarizing microscope images of *Zip13 OE* mouse liver after CCl4 treatment.** Livers from *Rosa-Cre^+/-^ Zip13OE ^+/-^* (*Zip13* overexpressed throughout the body) and control mice after 2 months of CCl_4_ gavage were examined. In the polarizing microscope images, as indicated by the white arrows, thick type I collagen fibers exhibit strong, bright red color, thinner type III collagen fibers appear green, whereas type IV collagens show yellow color. Scale bars, 200 μm.


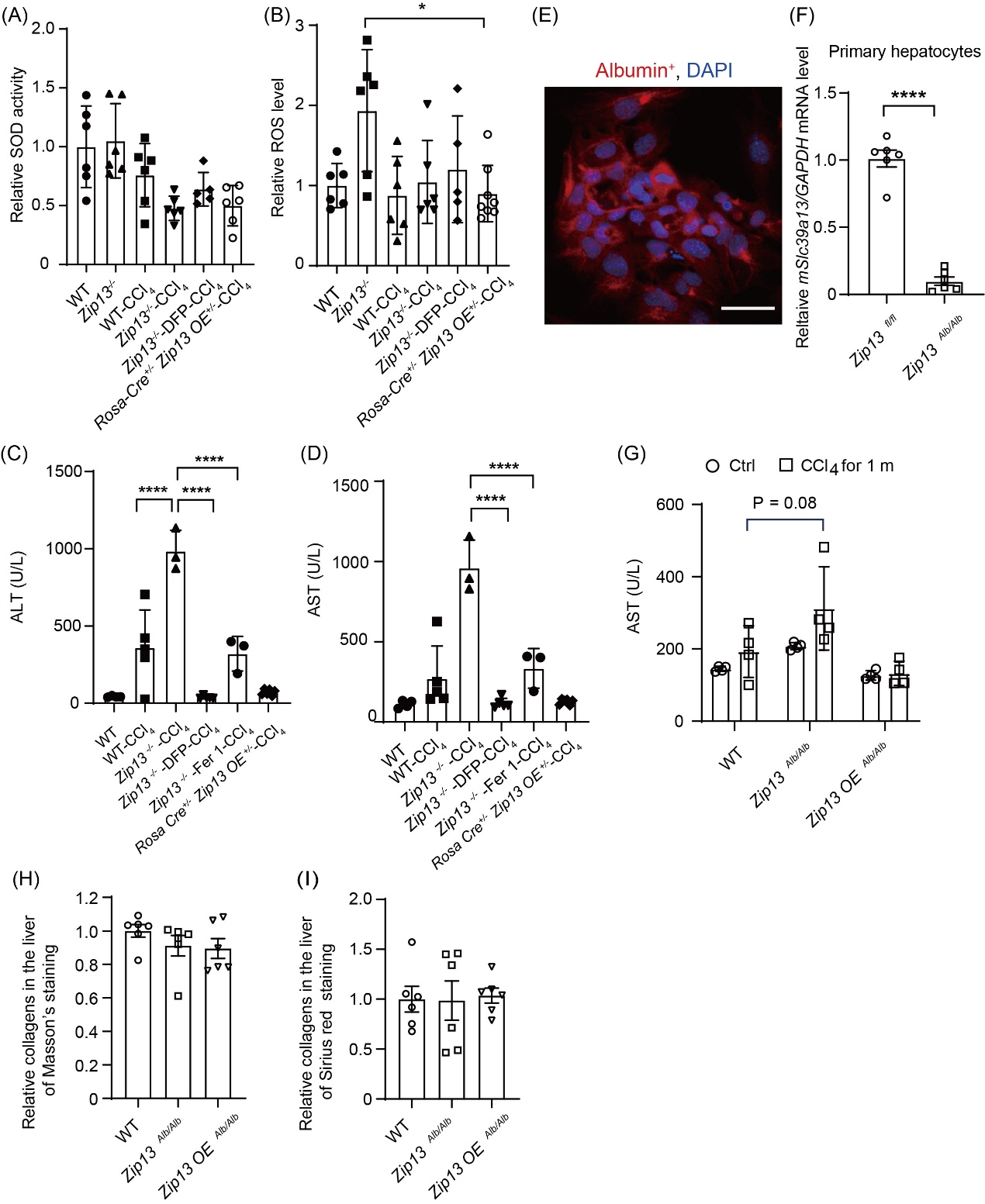


**Figure S5**. **ZIP13 deficiency exacerbates CCl_4_-liver toxicity via iron dyshomeostasis in hepatocytes.** Relative hepatic **(A)** SOD activity and **(B)** ROS levels in the liver, serum **(C)** ALT and **(D)** AST of untreated WT and *Zip13^-/-^* mice, and in mice induced by CCl_4_ for 1 month (WT, *Zip13^-/-^*, *Rosa-Cre^+/-^ Zip13OE ^+/-^*). Where indicated, deferiprone (DFP, 50 mg/kg, twice a week) in A-D, or ferrostain-1 (Fer-1, 5 mg/kg, twice a week) in C,D was intraperitoneally injected for 1 month during CCl_4_ treatment. n=6 in A and B, n=3-4 in C and D. **(E)** Immunofluorescence-mediated identification (albumin-ir hepatocytes were shown in red, with the nucleus stained blue (DAPI)), and **(F)** knockout efficiency of *Zip13* in hepatocytes isolated from *Zip13^fl/fl^* and *Zip13^Alb/Alb^* (*Zip13* knockout in hepatocytes) mice. n=6. **(G)** Serum AST, **(H,I)** quantitative analysis for collagens of liver sections by **(H)** Masson’s staining and **(I)** Sirius red staining in WT, *Zip13^Alb/Alb^* (*Zip13* knockout in hepatocytes) and *ZIP13 OE^Alb/Alb^* (*Zip13* overexpressed in hepatocytes) mice treated for 1 month with CCl_4_ gavage, n=4 in G, n = 6 in H and I. Scale bars: 50 μm in E. In A, B, C, D, F, G, H and I, the data are shown as the mean ± SEM. *, *P* < 0.05; ****, *P* < 0.001; ****, *P* < 0.0001. Statistical analysis in panels A, B, C, D, H and I was performed by the one-way ANOVA with multiple comparisons, in panels F and G by the two-sided Student’s t-test.


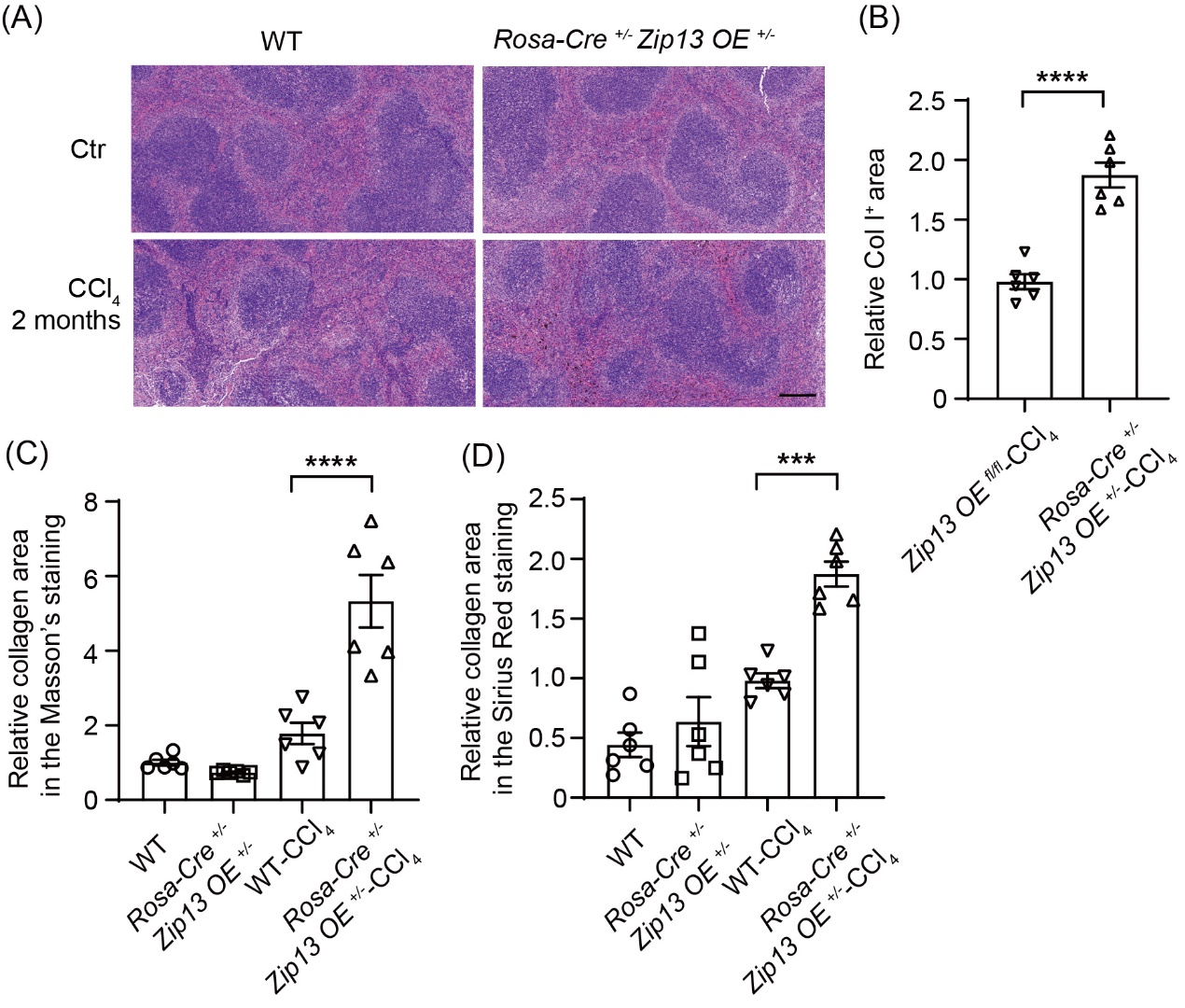


**Figure S6. Pathological analysis for the spleen and quantitative analysis for the staining of the liver sections of *Zip13 OE* mice after CCl4 treatment.** *Zip13 OE* (*Rosa-Cre^+/-^ Zip13OE ^+/-^*, *Zip13* overexpressed throughout the body) mice and control mice were treated with long-term (2 months) CCl_4_ treatment. **(A)** H&E staining for the spleen from the WT and *Rosa-Cre^+/-^ Zip13OE ^+/-^* (*Zip13* overexpressed throughout the body) mice with and without treatment (2 months) with CCl_4_. **(B)** Immuno-histochemical (IHC) staining (enhanced by DAB) for Col I in the liver of control (*Zip13 OE^fl/fl^*) and *Zip13 OE* (*Rosa-Cre ^+/-^ Zip13OE ^+/-^, Zip13* overexpressed throughout the body) mice following long-term (2 months) CCl_4_ treatment. n=6. **(C-D)** Quantification of **(C)** Masson’s staining and **(D)** Sirius red staining for the liver sections from the control and *Zip13 OE* (*Rosa-Cre^+/-^ Zip13OE ^+/-^*) mice with and without treatment (2 months) of CCl_4_. n=6. Scale bars in A, 100 μm. In B-D: the data are shown as the mean±SEM. ***, *P* < 0.001; ****, *P* < 0.0001. Statistical analysis in panel B was performed by the two-sided Student’s t-test, in C and D by the one-way ANOVA with multiple comparisons.


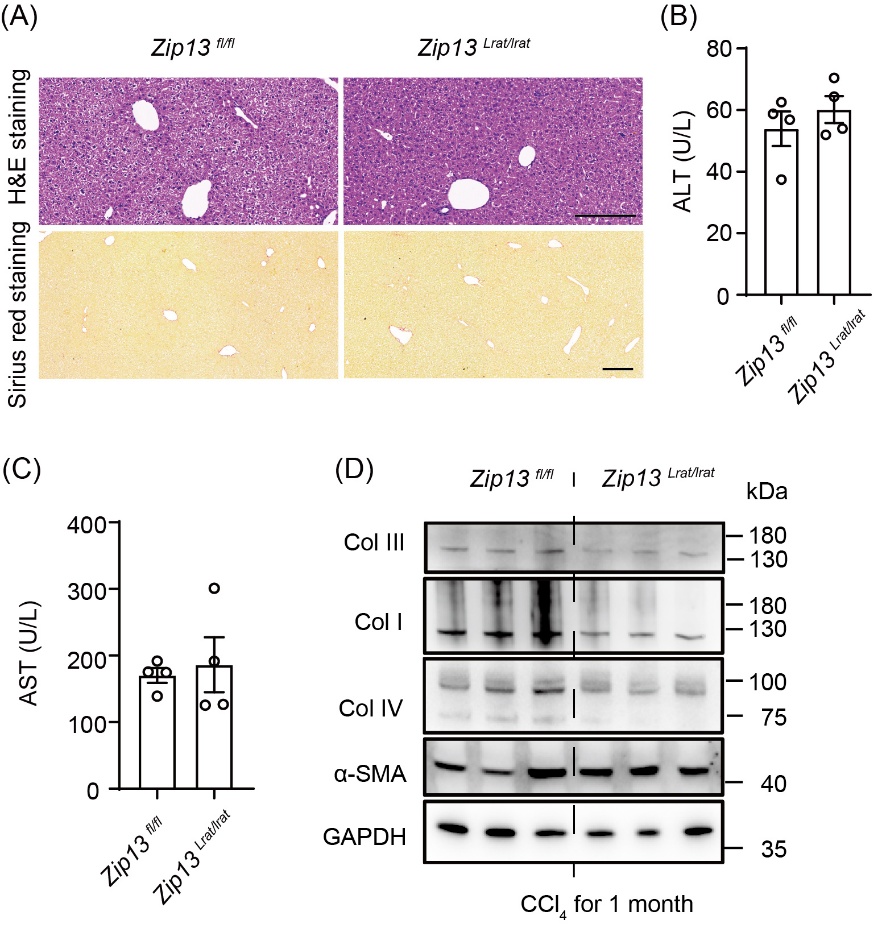


**Figure S7**. **ZIP13 deficiency in HSCs does not present obvious liver injury**. **(A)** Liver H&E and Sirius red staining, serum **(B)** ALT (n=4) and **(C)** AST (n=4) in untreated *Zip13^Lrat/Lrat^* (*Zip13* knockout in HSCs) and control *Zip13^fl/fl^* mice. **(D)** Western blotting results for hepatic Col I, III, IV, α-SMA and GAPDH from the *Zip13^Lrat/Lrat^* (*Zip13* knockout in HSCs) and *Zip13^fl/fl^* control mice after 1 month of CCl_4_ treatment. Scale bars, 200 μm in A. In B and C, data are shown as the mean ± SEM. Statistical analysis was performed by the two-sided Student’s t-test.


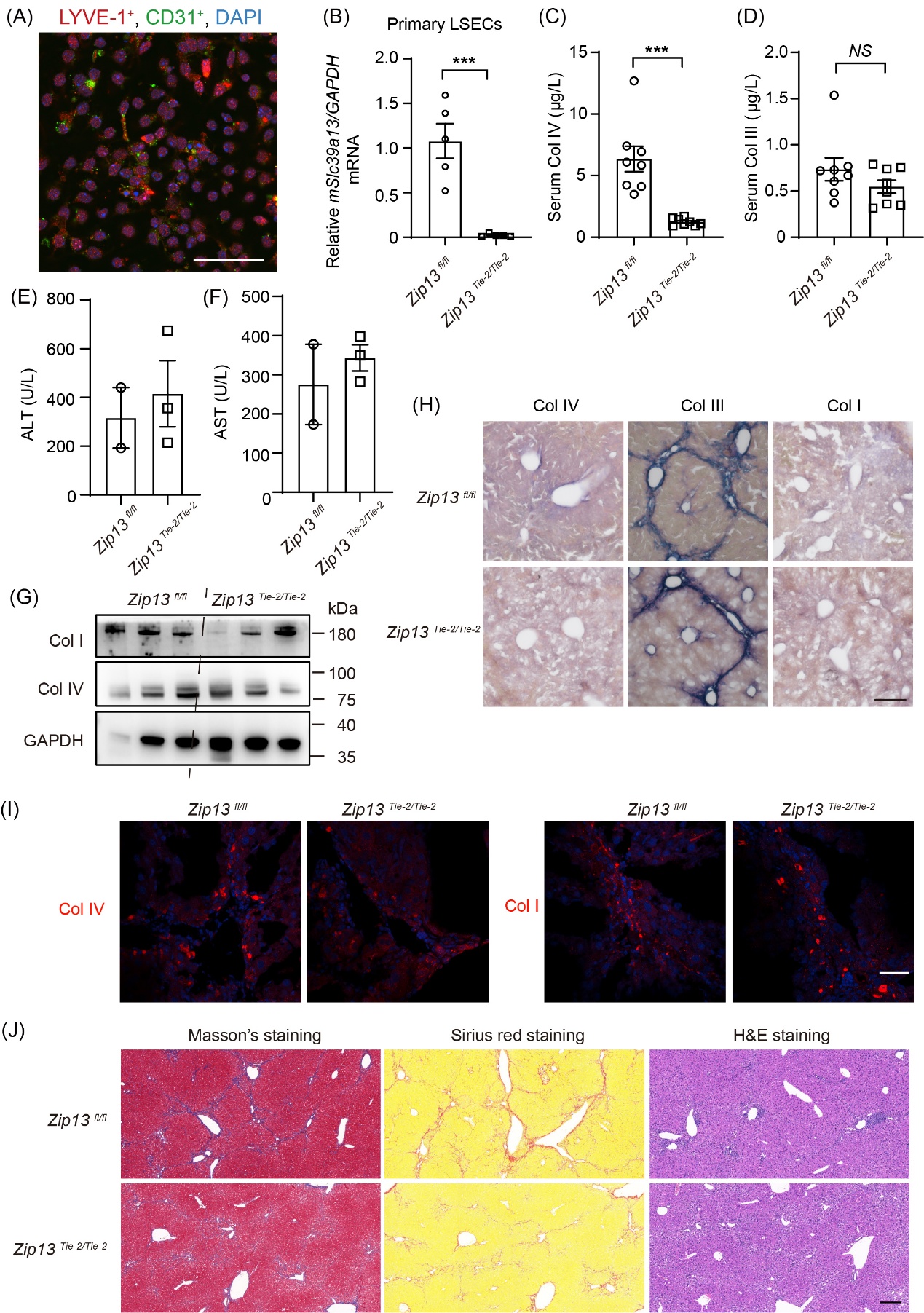


**Figure S8**. **ZIP13 loss in liver sinusoidal endothelial cells (LSECs) can only ameliorate the deposition of Col IV during liver fibrosis.** (A) Immunofluorescence-mediated identification of isolated LSECs (LYVE1^+^ LSECs were shown in red, CD31^+^ LSECs were shown in green, and the nucleus were stained blue (DAPI), and (B) knockout efficiency of *Zip13* in hepatocytes isolated from *Zip13^fl/fl^* and *Zip13^Tie-2/Tie-2^* (*Zip13* knockout in LSECs) mice. n=5. Serum (C) Col IV (n=8) and (D) Col III (n=8), (E) ALT (n=2-3), (F) AST (n=2-3), (G) Western blotting for hepatic Col I, Col III and Col IV, (H) IHC staining for hepatic Col IV, Col III and Col I enhanced by alkaline phosphatase substrate (BCIP/NBT, blue) (I) IF staining for hepatic Col IV (red) and Col I (red), and (J) Masson’s, Sirius red, H&E staining of the liver from *Zip13^fl/fl^* and *Zip13^Tie-2/Tie-2^* (*Zip13* knockout in LSECs) mice treated with CCl_4_ for 1 month. Scale bars: 50 μm in A and I, 200 μm in H and J. In B-F, data are shown as the mean ± SEM. *NS*, no significant; ***, *P* < 0.001. Statistical analysis was performed by the two-sided Student’s t-test.


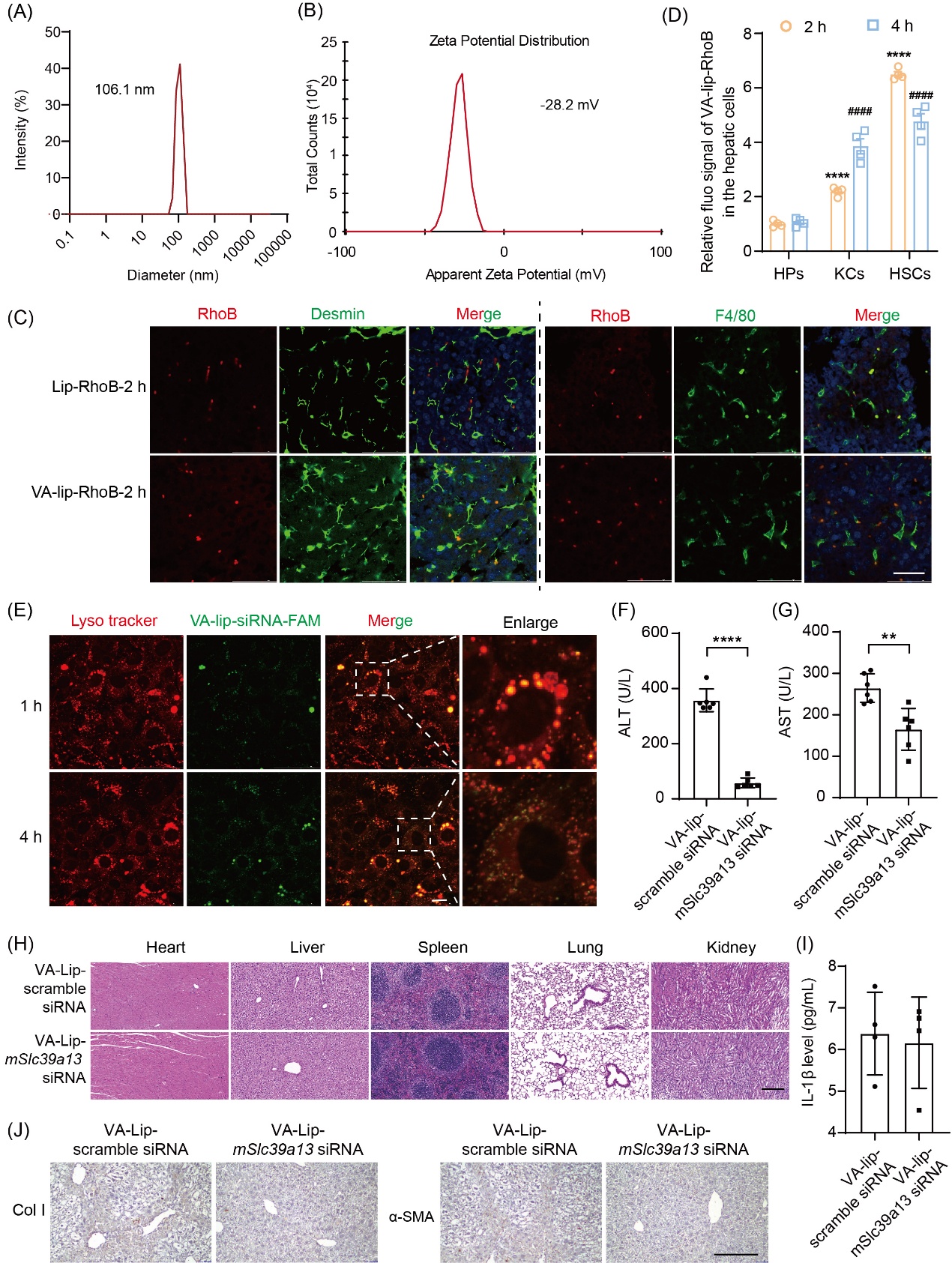


**Figure S9. Characterization of the VA-lip*-mSlc39a13* siRNAs, and their effects on the hepatotoxicity.** **(A,B)** Diameter and surface zeta potential for the VA-lip *mSlc39a13* siRNAs. **(C)** IF staining for desmin-immunoreactivity (desmin-ir) HSCs (green) and F4/80-ir Kupffer cells (green), and **(D)** quantitative analysis of relative VA-lip-RhoB in the hepatic cells (of desmin-ir HSCs, F4/80-ir Kupffer cells and albumin-ir hepatocytes) (measured by flow cytometry shown in Figure 7D) from the liver of wild-type (WT) mice intravenously injected with VA-lip-rhodamine B (VA-lip R, red) for 2 h and 4 h, respectively. ****, *P* < 0.0001, compared with signals in hepatocytes at 2 h; ####, *P* < 0.0001, compared with signals in hepatocytes at 4 h. Statistical analysis was performed by the two-sided Student’s t-test. **(E)** The localization of the siRNA-FAM (green) in the cytosolic compartments of microglial cells within 1 h or 6 h of treatment. **(F)** Serum ALT and **(G)** AST following administration of VA-lip mSlc39a13 siRNAs in mice treated for 3 weeks with CCl_4_. n=6. **(H)** H&E staining of the heart, liver, spleen, lung and kidney and **(I)** plasma IL-1β levels (n=4) from mice administered VA-lip mSlc39a13 siRNAs. **(J)** IHC staining for Col I and α-SMA enhanced by DAB of liver sections from mice injected with VA-lip mSlc39a13 siRNA, under CCl4 treatment. Scale bars, 50 μm in C, 20 μm in D, 200 μm in G and I. In D, F, G and I, data are shown as the mean±SEM. **, *P* < 0.01; ****, *P* < 0.0001. Statistical analysis in panels F, G and I was performed by the two-sided Student’s t-test.

**
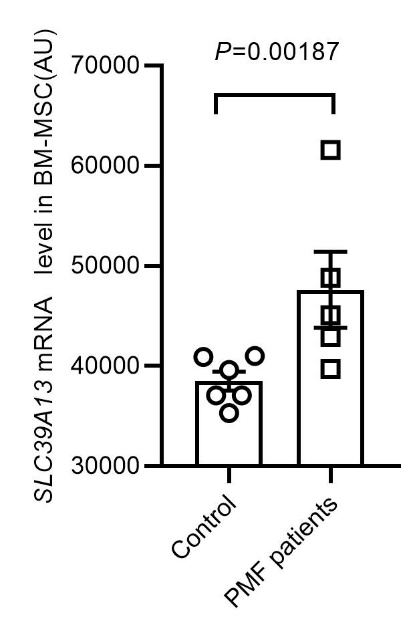
**

**Figure S10. *SLC39A13* mRNA levels in the** **bone marrow mesenchymal stromal cells (BM-MSC) from healthy controls and patients with primary myelofibrosis (PMF).** This result was generated from the database (GSE44426) in the publication [1]. n=6 in control group, n=5 in PMF patients. Data are shown as the mean ± SEM. For this RNA sequencing result, statistical analysis was performed using the algorithm of Benjamini & Hochberg test.

**Reference**

[1] C. Desterke, C. Martinaud, B. Guerton, L. Pieri, C. Bogani, D. Clay, F. Torossian, et al, “Tetraspanin CD9 participates in dysmegakaryopoiesis and stromal interactions in primary myelofibrosis,” *Haematologica* **2015**, *100* (6), 757, https://doi.org/10.3324/haematol.2014.118497.
